# Bi_12_Rh_3_Ag_6_I_9_: Mobile Silver Ions and Surface-Dominated Transport in a Layered Weak Topological Insulator

**DOI:** 10.1021/acs.inorgchem.6c02517

**Published:** 2026-10-06

**Authors:** Eduardo Carrillo-Aravena, Johannes Heßdörfer, Christian N. Saggau, Armando Consiglio, Shailja Sharma, Friedrich Reinert, Giorgio Sangiovanni, Domenico Di Sante, Oleg Janson, Kornelius Nielsch, Nicolás Pérez, Michael Ruck

**Affiliations:** † Faculty of Chemistry and Food Chemistry, Dresden University of Technology, 01062 Dresden, Germany; ‡ Würzburg-Dresden Cluster of Excellence ctd.qmat, Dresden University of Technology, 01062 Dresden, Germany; § Faculty of Physics and Astronomy, University of Würzburg, 97074 Würzburg, Germany; ∥ Leibniz Institute for Solid State and Materials Research, 01069 Dresden, Germany; ⊥ Department of Electrical and Photonics Engineering, 5205 Technical University of Denmark, 2800 Kgs. Lyngby, Denmark; # Center for Silicon Photonics for Optical Communications (SP.O.C), Technical University of Denmark, 2800 Kgs. Lyngby, Denmark; ¶ Department of Physics and Astronomy, 9296 University of Bologna, 40127 Bologna, Italy; ∇ Center for Computational Quantum Physics, Flatiron Institute, New York, New York 10010, United States; ○ Institute of Applied Physics, Dresden University of Technology, 01062 Dresden, Germany; ⧫ Institute of Materials Science, Dresden University of Technology, 01062 Dresden, Germany

## Abstract

Bi_12_Rh_3_Ag_6_I_9_ extends the Bi_14_Rh_3_I_9_ family of layered bismuth-rich iodides in which intermetallic topological-insulator sheets alternate with salt-like spacer layers. High-temperature synthesis yields phase-pure powders and millimeter-sized platelets. Single-crystal X-ray diffraction reveals a rhombohedral structure composed of [Bi_12_Rh_3_I]^2+^ layers and [Ag_6_I_8_]^2–^ spacers. In this spacer, Ag^+^ cations occupy 75% of the tetrahedral voids of a double iodide layer, giving rise to intrinsic two-dimensional cation disorder and mobility. Electrochemical impedance spectroscopy reveals a room-temperature ionic conductivity of about 10^–6^ S cm^–1^. Density-functional theory including spin–orbit coupling predicts band inversion and weak-topological-insulator invariants, while angle-resolved photoemission spectroscopy corroborates the calculated bulk electronic structure, yielding an experimental bulk band gap of 286 meV. Transport measurements show strongly anisotropic, initially *n*-type conduction. During the initial measurement cycle, applied bias reduces the electron doping through slow electrochemical deintercalation of silver. After conditioning, bulk conduction is strongly reduced; transport below about 130 K becomes nonactivated, the bias-induced drift ceases below about 50 K as Ag^+^ motion freezes, and a pronounced resistivity drop perpendicular to the layers below 3.2 K is suggestive of a boundary-sensitive low-dimensional transport regime. Bi_12_Rh_3_Ag_6_I_9_ demonstrates that spacer chemistry provides a chemical lever for carrier-density control in layered topological insulators.

## Graphical abstract

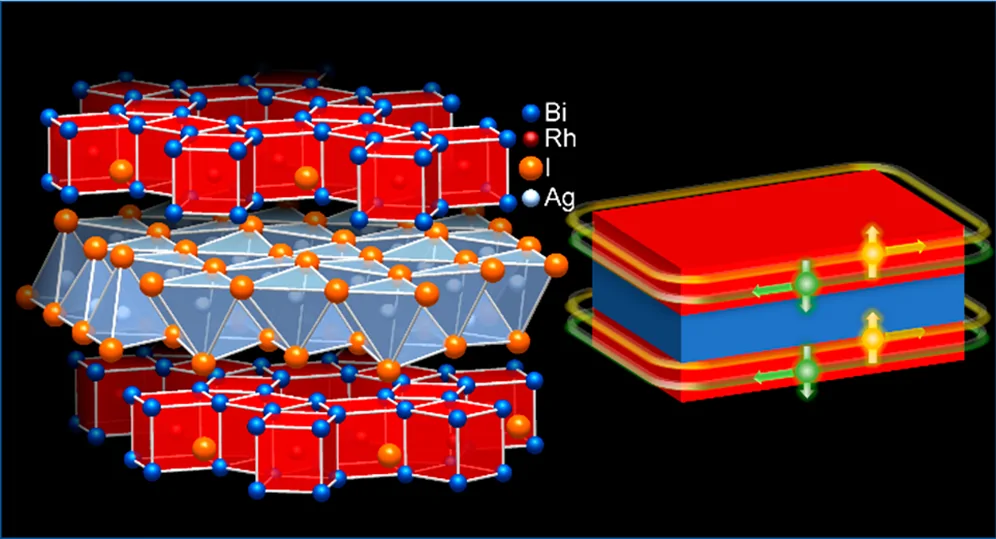

## Introduction

Topological insulators combine a bulk energy gap with gapless, spin-momentum–locked boundary states protected by time-reversal symmetry.^1–6^ In weak topological insulators (WTIs), these states occur only on selected crystal faces and, in the generic layer construction, arise from stacked 2D topological insulators separated by topologically trivial insulating spacers (Figure 1a).^4^ This description in terms of stacked layers is particularly appealing from a chemical perspective because it enables modular materials design by changing the spacer layer while retaining the electronic motif responsible for band inversion.

**Figure 1:**
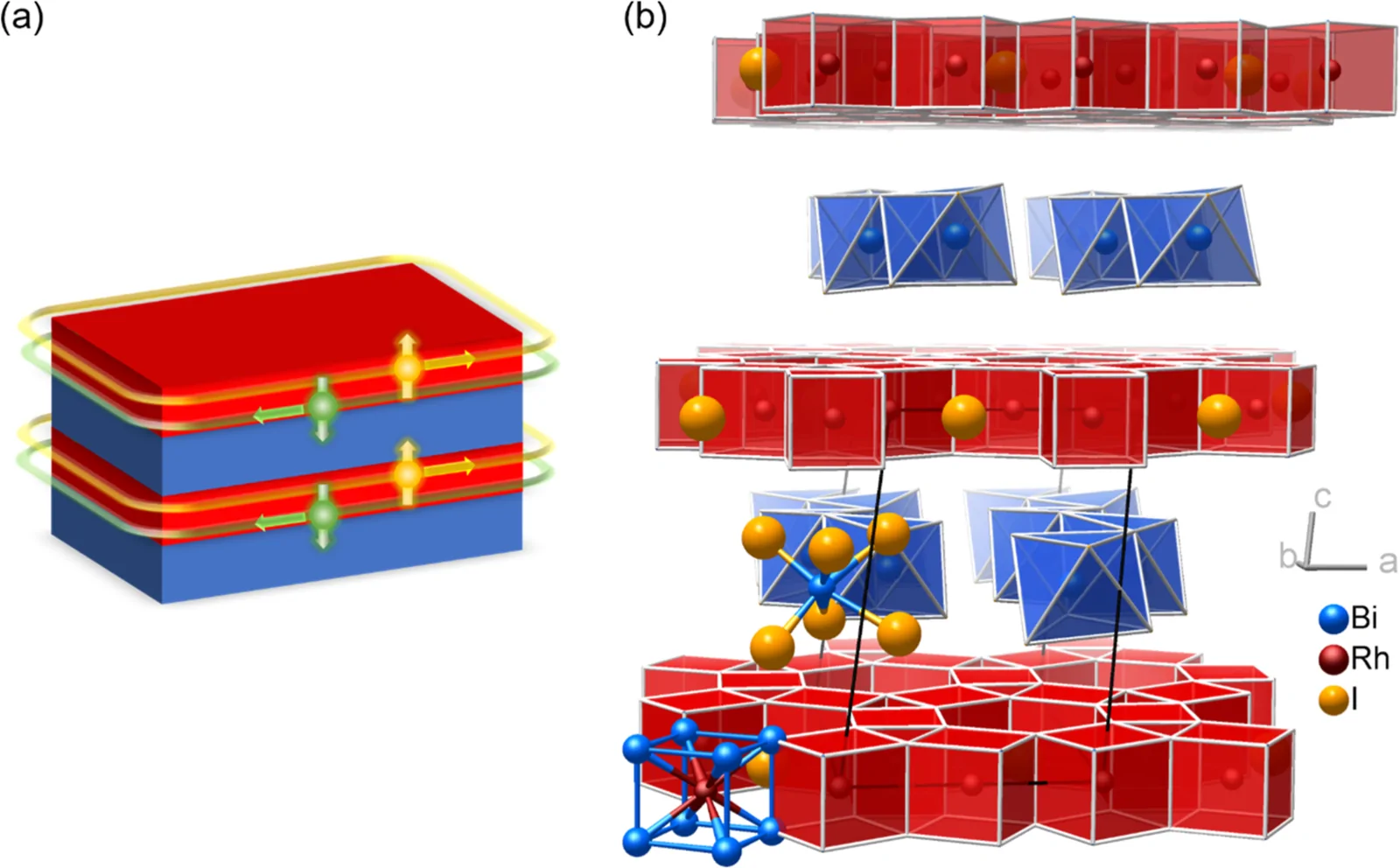
(a) Generic construction of a WTI as a stack of 2D TIs (red) that are spatially and electronically separated by topologically trivial insulating spacers (blue). Topological boundary states occur on side facets exposing the edges of the 2D TI layers. In these states, electrons are protected against backscattering and the electron spin is locked orthogonal to the propagation vector. (b) Crystal structure of Bi_14_Rh_3_I_9_, the first realization of this crystalline WTI family. The [(Bi_12_Rh_3_)­I]^2+^ layer carries the quantum-spin-Hall band-inversion motif, whereas the salt-like [Bi_2_I_8_]^2–^ chains separate the topological layer.

Bi_14_Rh_3_I_9_ is a prototypical WTI that can be described as a layered salt built from intermetallic [(Bi_12_Rh_3_)­I]^2+^ sheets (ruby/kagome-type) separated by [Bi_2_I_8_]^2–^ spacers (Figures 1b and S1).^7–10^ The intermetallic sheet exhibits a graphene-like Dirac cone at the *K* point that opens a sizable gap when spin–orbit coupling is included. Theoretical calculations have shown that modifying the spacer layer and/or the transition metal tunes the bulk band gap while preserving strong charge separation between the layers. As a result, the bulk retains the essential band-structure features of the electronically decoupled layers, including the spin–orbit-driven band inversion discussed previously.^9,10^ This chemistry-led heterostructure concept has enabled the discovery of a series of related compounds with modified spacers, transition-metal substitutions, or altered layer stacking (Figure S1), providing a route to tune interlayer coupling and the topological gap.^11–15^

Local transport studies on Bi_14_Rh_3_I_9_ revealed clearly distinct transport characteristics of the spacer layer, the intermetallic layer and, notably, the edge channels.^9^ Likewise, indenter-type microprobe measurements on Bi_12_Rh_3_Sn_3_I_9_ and Bi_12_Rh_3_Cu_2_I_5_ showed length-independent sheet resistance and approximately activated transport behavior upon cooling.^12,13^

A recurring practical issue in this compound family is the control of bulk carrier density. Charge balance at crystal terminations and iodine loss under vacuum can shift the chemical potential into the conduction band (n-doping), which is undesirable when surface or edge transport is targeted. Here we introduce a different chemical lever: an ion-mobile spacer that can act as an internal dopant reservoir. Bi_12_Rh_3_Ag_6_I_9_ can be written as [Bi_12_Rh_3_I]­[Ag_6_I_8_] and replaces the rigid bismuth-iodide spacer by an AgI-derived double layer containing partially occupied tetrahedral sites. Because silver can be electrochemically extracted (or inserted), the electronic doping level becomes chemically adjustable without changing the intermetallic framework.

In this first report on Bi_12_Rh_3_Ag_6_I_9_, we focus on synthesis, crystal chemistry and the bulk electronic structure, and we present an initial set of bulk transport measurements that establish the basic transport anisotropy and identify low-temperature phenomena that motivate more detailed future work.

## Results and Discussion

### Synthesis and Phase Stability

Temperature profiles were guided by protocols established for Bi_14_Rh_3_I_9_.^7^ Phase-pure microcrystalline Bi_12_Rh_3_Ag_6_I_9_ samples and black platelet crystals (typical lateral size up to several millimeters) were obtained from sealed-ampoule reactions of Bi, Rh, Ag, and BiI_3_ (Figure S2). Differential scanning calorimetry and ex situ annealing experiments show that the target phase starts to form once the starting mixture is heated above 261 °C, close to the eutectic in the Bi–I system, but its formation is kinetically hindered (Figure S3). Mechanical pre-activation (ball milling) is essential for accessing single-phase powders reproducibly. Single-phase products are obtained after annealing in the range 345–460 °C, whereas decomposition occurs at 479(2) °C into Bi, ht-RhBi_2_, AgI, and BiI_3_. More details on synthesis optimization and crystal growth, as well as powder diffractograms and EDX analyses, can be found in the Supporting Information (Figures S2–S8). Bi_12_Rh_3_Ag_6_I_9_ can be handled in air without visible decomposition, but should be stored under inert gas. In a vacuum, the compound loses some iodine over time.

### Crystal Structure

Single-crystal X-ray diffraction at 296 and 100 K reveals a rhombohedral structure (*R*3̅*m*, hexagonal setting, *a* = 9.1585(4) Å, *c* = 38.946(2) Å at 296 K) with three alternating intermetallic and spacer layers per unit cell (Figure 2, Tables S1–S4). As in Bi_14_Rh_3_I_9_, the intermetallic layer [(Bi_12_Rh_3_)­I]^2+^ is built from edge-sharing, rhodium-centered bismuth cubes (Rh–Bi 2.8299(2)–2.8353(2) Å at 296 K) that form a prismatic rhombitrihexagonal honeycomb. Equivalently, the arrangement can be described as a kagome net when focusing on the rhodium atoms, or as a ruby lattice when emphasizing the bismuth framework. Each hexagonal prismatic void hosts an iodide ion at its center (Bi–I1 3.47–3.52 Å at 296 K).

**Figure 2:**
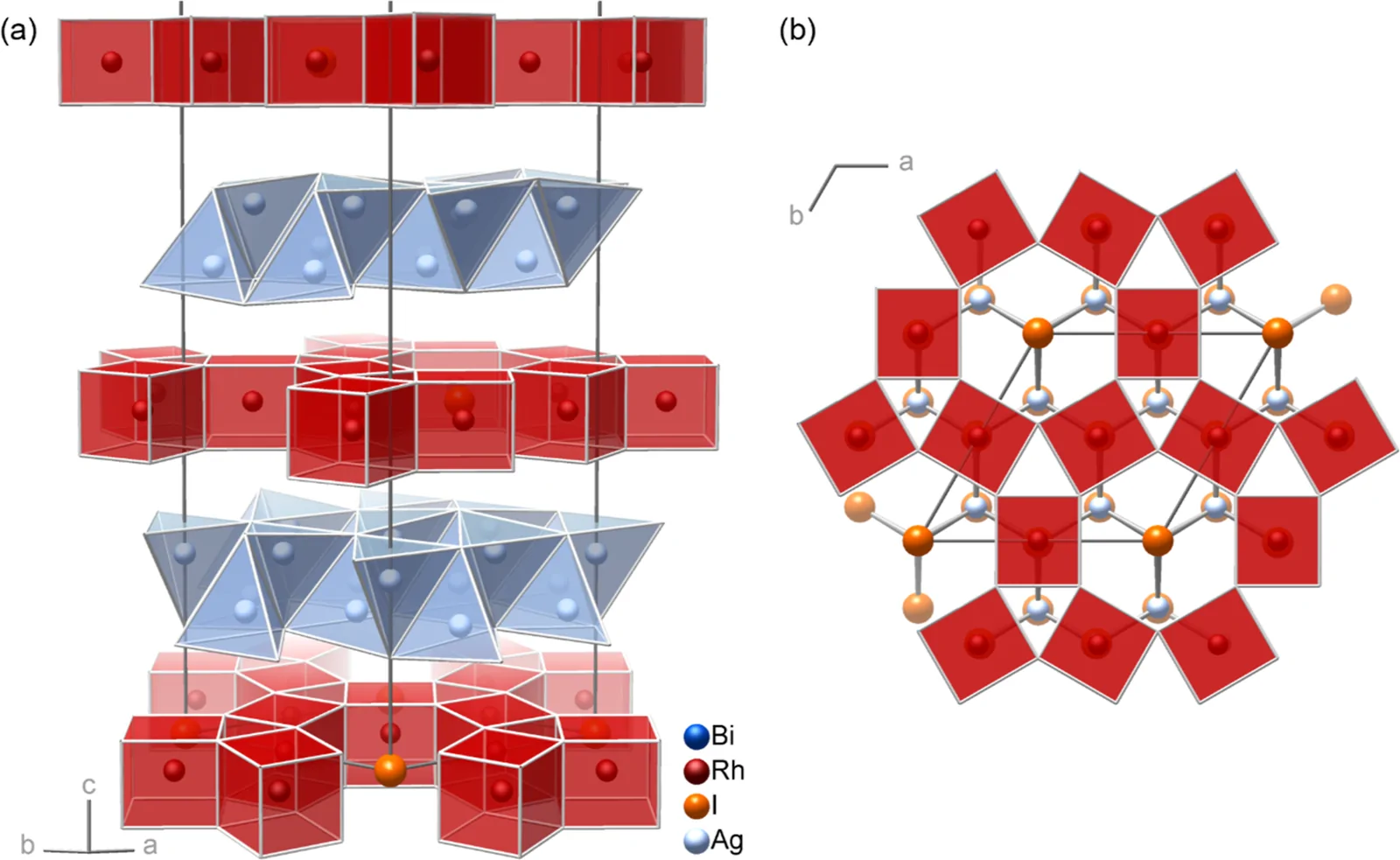
Crystal structure of Bi_12_Rh_3_Ag_6_I_9_. (a) Nets of edge-sharing rhodium-centered bismuth cubes (red) with iodide ions in their hexagonal voids constitute the intermetallic layer [(Bi_12_Rh_3_)­I]^2+^. Edge-sharing silver-centered iodide tetrahedra (blue) form the [Ag_6_I_8_]^2–^ spacer. About two-thirds of the rhombohedral unit cell in *c*-direction are shown. (b) Top view of a [(Bi_12_Rh_3_)­I]^2+^ layer and the underlying [Ag_6_I_8_]^2–^ spacer layer. The average occupancy of the silver atoms in the tetrahedral voids is 75%.

The [Ag_6_I_8_]^2–^ spacer is derived from a double layer of hexagonally packed iodide ions. Six Ag^+^ cations per formula unit occupy the eight available tetrahedral voids, corresponding to an average occupancy of 75%. The pronounced change in the silver distribution between room temperature and 100 K—from a more homogeneous distribution at room temperature to preferential occupation of the Ag2 site at 100 K—provides clear evidence for Ag^+^ mobility. Ag–I distances (2.804(2)–2.924(2) Å at 296 K) closely match those in wurtzite- and sphalerite-type AgI (β-AgI, 2.797–2.824 Å; γ-AgI, 2.814 Å; Figure S9).^16,17^ Since the layer groups of the intermetallic layer (*p*6/*mmm*, no. 80) and of the spacer (*p*3̅*m*1, no. 72) as well as their stacking match common threefold axes, the space group symmetry is considerably higher than for all other members of the structure family. The orthogonal distance between the top and bottom layers of two adjacent intermetallic layers is 981 pm (with respect to atom centers).

Bi_12_Rh_3_Ag_6_I_9_ crystals show a tendency toward reverse-obverse twinning and stacking disorder, which gives rise to diffuse intensity along reflection rows in the c* direction. This disorder affects the local stacking of spacer layers and may include occasional intergrowth of closely related spacer variants, in particular the one-atom-thick [Ag_2_I_4_]^2–^ spacer found in the related compound Bi_12_Rh_3_Ag_2_I_5_ and, less frequently, the [Bi_2_I_8_]^2–^ spacer known from Bi_14_Rh_3_I_9_. The single-crystal used for the structure refinement was selected for minimal diffuse scattering and twinning; the refined model therefore represents the average structure of the dominant Bi_12_Rh_3_Ag_6_I_9_ stacking sequence. The residual electron density in the spacer region is consistent with this local stacking disorder and the positional disorder of mobile Ag^+^ cations.

### Bulk Electronic Structure

The Kane and Mele model^1^ provides the conceptual framework for understanding the quantum spin Hall effect in the intermetallic layer, whose structural symmetry is analogous to that of graphene.^10^ This is reflected in the scalar-relativistic electronic structure (Figure 3a), which shows a band crossing at the *K* point close to the Fermi level (*E*_F_). The corresponding states are dominated by the intermetallic [(Bi_12_Rh_3_)­I]^2+^ layer, with major contributions from Bi 6p states and Rh 4d admixture. When spin–orbit coupling (SOC) is included (Figure 3b–e), the degeneracy at the *K* point is lifted and a calculated band gap of about *E*_g_ = 240 meV opens. The large SOC of Bi is therefore essential for the non-trivial electronic structure, in analogy to the established Bi_14_Rh_3_I_9_. Also in the fully relativistic band structure of Bi_12_Rh_3_Ag_6_I_9_, the uppermost valence bands and the lowest conduction bands derive predominantly from the intermetallic layers. The weak overlap of the charge-density isosurfaces of the individual layers (Figure S10) is consistent with the pronounced ionic separation between intermetallic layer and spacer.

**Figure 3:**
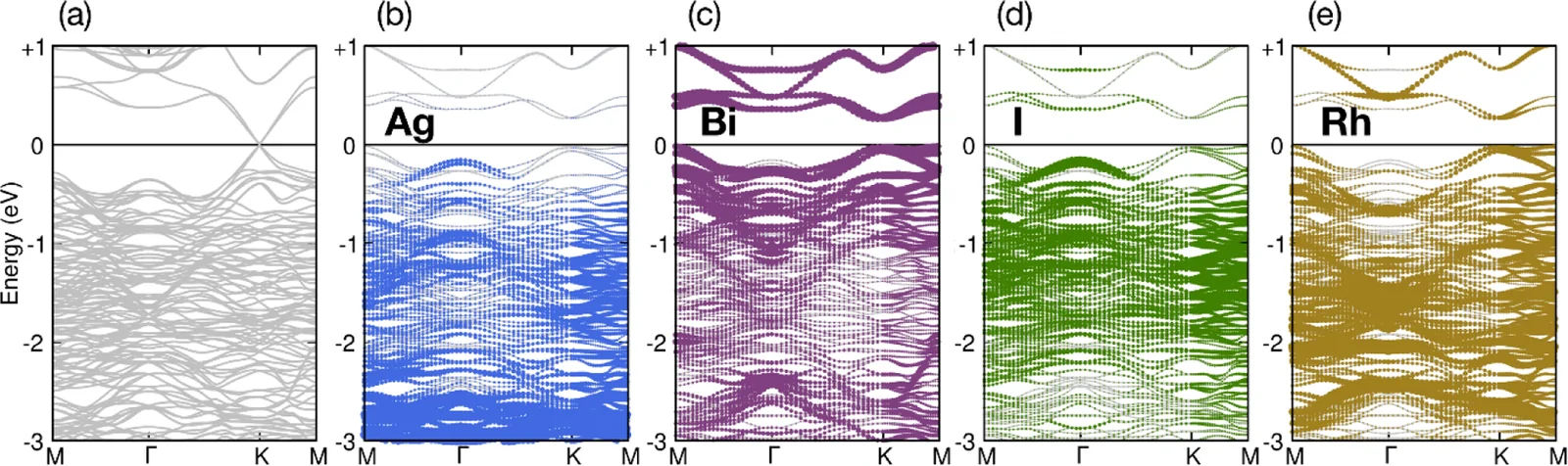
Calculated bulk band structure of Bi_12_Rh_3_Ag_6_I_9_: (a) scalar-relativistic (without spin–orbit coupling) and (b–e) fully relativistic (including spin–orbit coupling) calculations. The element-specific contributions are indicated by the color coding. The band structure was calculated on an ordered structural approximant. This approximation mainly concerns the ion-mobile spacer; the SOC-induced gap and weak-topological classification are dominated by the Bi and Rh states of the intermetallic framework.

The layered electronic structure of Bi_12_Rh_3_Ag_6_I_9_ corresponds to the generic construction of a weak topological insulator as a three-dimensional crystal that is adiabatically connected to a stack of two-dimensional quantum-spin-Hall insulators separated by topologically trivial insulating spacers.^18^ In this sense, the intermetallic [(Bi_12_Rh_3_)­I]^2+^ layer represents the electronically active 2D-TI building block, whereas the salt-like spacer electronically separates the layers and controls interlayer coupling, band alignment, and carrier density. This layer-resolved description is inspired by artificial topological heterostructures^2,3^ and well established for the Bi_14_Rh_3_I_9_ family: Bi_14_Rh_3_I_9_ was identified as a crystalline WTI built from such stacked topological layers,^8^ STM/STS directly revealed robust topological edge channels at steps of the cationic layer,^9^ and subsequent calculations showed that the isolated intermetallic [(Bi_12_Rh_3_)­I]^2+^ sheet is a 2D TI whose nontrivial band character is governed by the Bi/Rh-derived electronic structure of the intermetallic layer.^10^ The term “weak” refers to the weak *Z*_2_ indices and the associated stacking vector, not to an absence or weakness of topological protection.^19^

Formally, however, the topological invariant of the actual bulk crystal is not obtained by assigning independent parity products to isolated structural fragments. It is calculated from the Bloch wave functions of the complete periodic three-dimensional structure. Because Bi_12_Rh_3_Ag_6_I_9_ possesses inversion symmetry, the *Z*_2_ invariants can be determined from the parities of the occupied states at the time-reversal-invariant momenta of the full Brillouin zone.^20^ For Bi_12_Rh_3_Ag_6_I_9_, the invariants with respect to the R-centered hexagonal unit cell are *Z*_2_ = (0; 0, 0, 1), identifying the compound as a weak topological insulator with the same topology as the well-established and experimentally confirmed Bi_14_Rh_3_I_9_. The final weak index corresponds to the stacking direction and implies that topological boundary states are expected on side facets exposing the edges of the intermetallic 2D-TI layers, whereas the basal (001) surface is topologically trivial.

X-ray photoelectron spectroscopy (XPS) and angle-resolved photoemission spectroscopy (ARPES) on freshly cleaved (001) surfaces confirm the composition and reveal valence-band dispersions that closely follow the calculated bulk band structure (Figure 4). The samples typically develop n-type band bending and/or mild electron doping under vacuum, shifting the chemical potential upward and thereby enabling photoemission access to the conduction-band minimum. A leading-edge analysis^21^ yields a bulk band gap of *E*_g_ = 286 meV at the *K* point, corroborating the calculated band structure and establishing Bi_12_Rh_3_Ag_6_I_9_ as the widest-gap member of this structural family reported to date. The weak spectral weight observed within the gap originates from the polar termination of the layered salt and should not be confused with topological surface states. Consistent with the weak-topological classification *Z*_2_ = (0; 0,0,1), the basal (001) faces of the platelet crystals are not expected to host topological surface states. Access to the in-gap topological surface states on the lateral facets is, however, precluded in the present samples because the edge faces are both small and nonplanar, providing insufficient well-defined surface area for ARPES.

**Figure 4:**
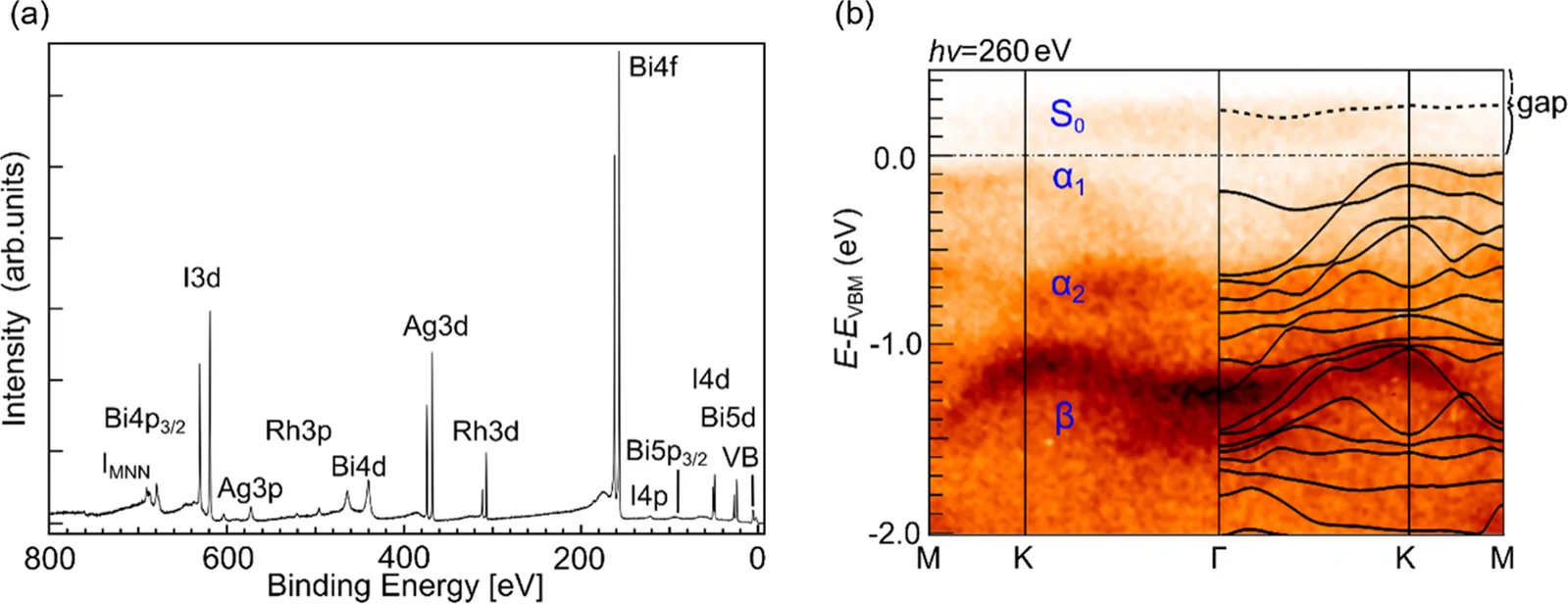
(a) XPS spectrum of a cleaved Bi_12_Rh_3_Ag_6_I_9_ crystal (*h*ν = 1200 eV, *T* = 30 K, Shirley-type background corrected, taken at Petra III). All peaks are marked by their respective core level, and there is no sign of a contamination by foreign elements discernible at this scale. (b) ARPES spectrum on the (001) crystal face (*h*ν = 260 eV, *T* = 30 K, cleaved at low temperature), measured along the indicated high-symmetry direction. The high symmetry points are indicated by vertical solid lines. The energy is referenced to the bulk VB maximum (dot-dash line). Main features are marked by Greek letters. Overlaid is the calculated band structure of a [Bi_12_Rh_3_I]^2+^ layer. The dashed in-gap band is a feature of the surface projection and is sensitive to the degree of charge compensation. This band also hints the intermetallic layer termination as a source of the observed surface state S_0_.

### Ionic Conductivity

The partially occupied tetrahedral silver sites in the spacer layer suggest Ag^+^ mobility, analogous to AgI.^16,17^ Consistent with this picture, at room temperature the residual electron density associated with the silver positions is markedly delocalized and cannot be represented adequately by a simple displacement ellipsoid (Figure 5). The corresponding isosurfaces exhibit pronounced protrusions directed toward the trigonal faces of the surrounding tetrahedra, i.e., along the expected low-barrier pathway for Ag^+^ hopping between adjacent voids. Supporting this interpretation, single-crystal X-ray diffraction data collected at 100 K on the same specimen reveal a distinctly altered silver distribution. Moreover, the electron density at the silver sites becomes substantially more localized and essentially spherical, consistent with a reduced positional disorder upon cooling and thus with thermally activated Ag^+^ mobility at higher temperature.

**Figure 5:**
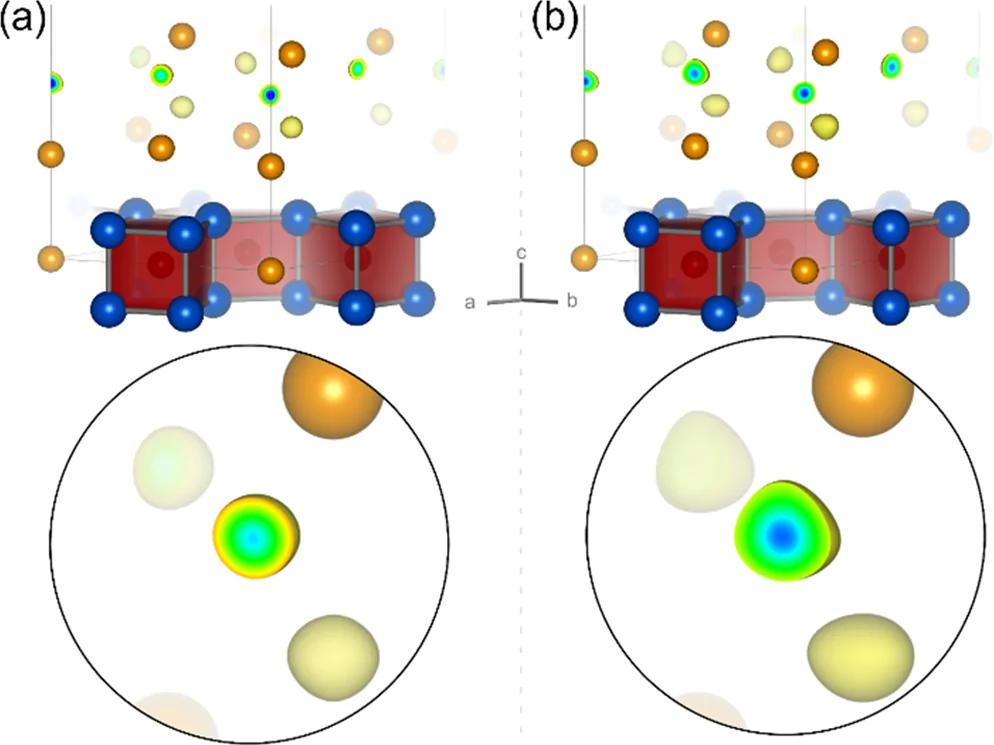
Fourier map showing electron density at the silver atom positions at 100 K (a) and room temperature (b) in a general view (top) and in detail (bottom). The shape of the electron density, especially at room temperature, is poorly described by harmonic oscillations.

Electrochemical impedance spectroscopy on a cold-pressed, sintered pellet reveals an ionic conductivity of approximately 10^–6^ S cm^–1^ at room temperature (Figure 6). Although this value is far below that of α-AgI—an outstanding Ag^+^ conductor above 146 °C—it is unexpectedly high for a structurally confined, quasi-two-dimensional ion-transport pathway and such a high occupancy of the available cation sites. Notably, comparable room-temperature conductivities have been reported for bulk γ-AgI,^22^ even though γ-AgI provides a higher density of accessible vacancies and allows three-dimensional Ag^+^ migration. In Bi_12_Rh_3_Ag_6_I_9_, the ionic transport is sufficient to enable slow electrochemical deintercalation under an applied bias (see below), offering a practical route to compensate inadvertent n-type doping. It is plausible that the measured ionic conductivity includes contributions not only from Ag^+^ transport through the bulk spacer network, but also from enhanced mobility in near-surface and grain-boundary space-charge regions of the crystallites forming the pellet.

**Figure 6:**
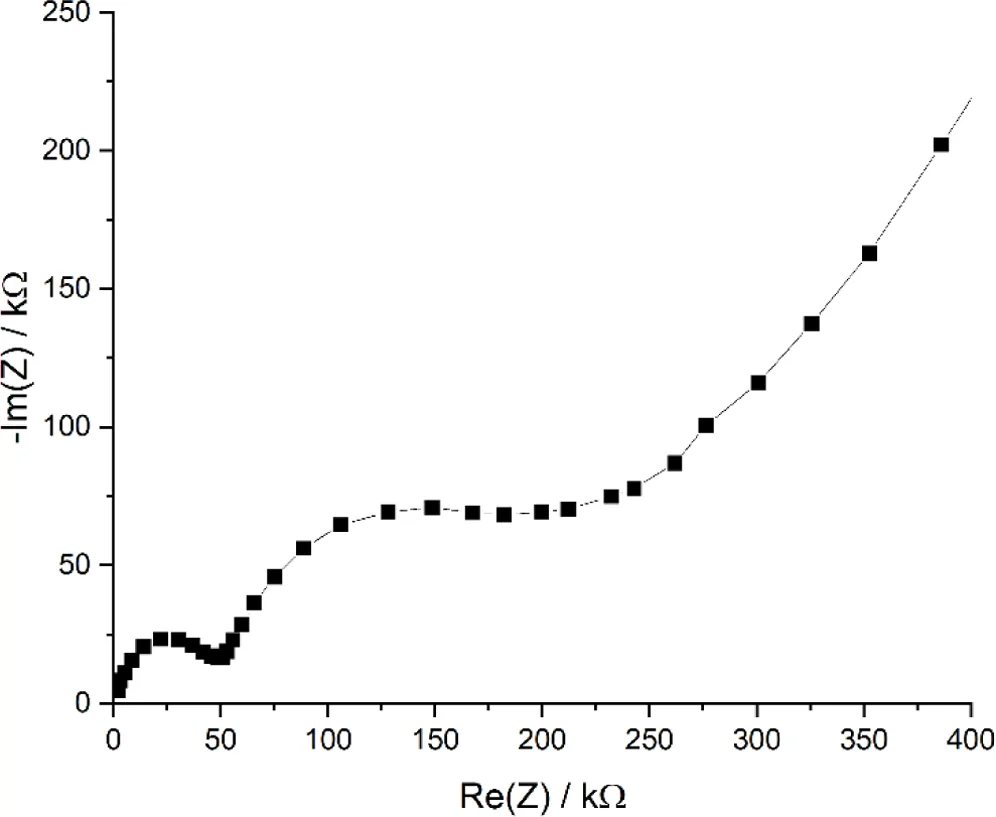
Nyquist impedance plot of a pressed pellet of Bi_12_Rh_3_Ag_6_I_9_. The two capacitances at high frequencies attributed to ionic conductivity and the grain boundary capacitance were used to calculate the ionic fractions of the conductivity.

### Electronic Transport

The two-point resistance of a small Bi_12_Rh_3_Ag_6_I_9_ crystal measured parallel to the *ab* planes shows a pronounced first-cycle evolution that depends on both temperature and the duration of current flow (Figure 7b). Above 50 K, the resistance recorded on heating is significantly higher than that measured during the preceding cooling branch, whereas this irreversibility is largely absent in subsequent thermal cycles. In the first cooling run, the resistance decreases continuously from 50 K down to 1.8 K and therefore does not show the activated increase expected for an ordinary semiconductor in this temperature range. Upon reheating, the resistance passes through a broad maximum near 130 K. The same characteristic temperature is recovered in four-point measurements on a previously cycled crystal (Figures 8a and S11), indicating that it marks the onset of a stabilized transport regime after the initial current and temperature treatment. A related deviation from simple thermally activated bulk transport was previously observed for Bi_12_Rh_3_Cu_2_I_5_, where the resistance showed a semiconductor-like increase upon cooling but became nearly temperature-independent below about 50 K in local transport experiments.^13^ Together with scanning tunneling microscopy results on Bi_14_Rh_3_I_9_, which established clearly different transport characteristics for the spacer, intermetallic layer, and edge channels,^9^ these observations suggest that charge transport in this compound family cannot be described by a single homogeneous bulk-semiconductor channel.

**Figure 7:**
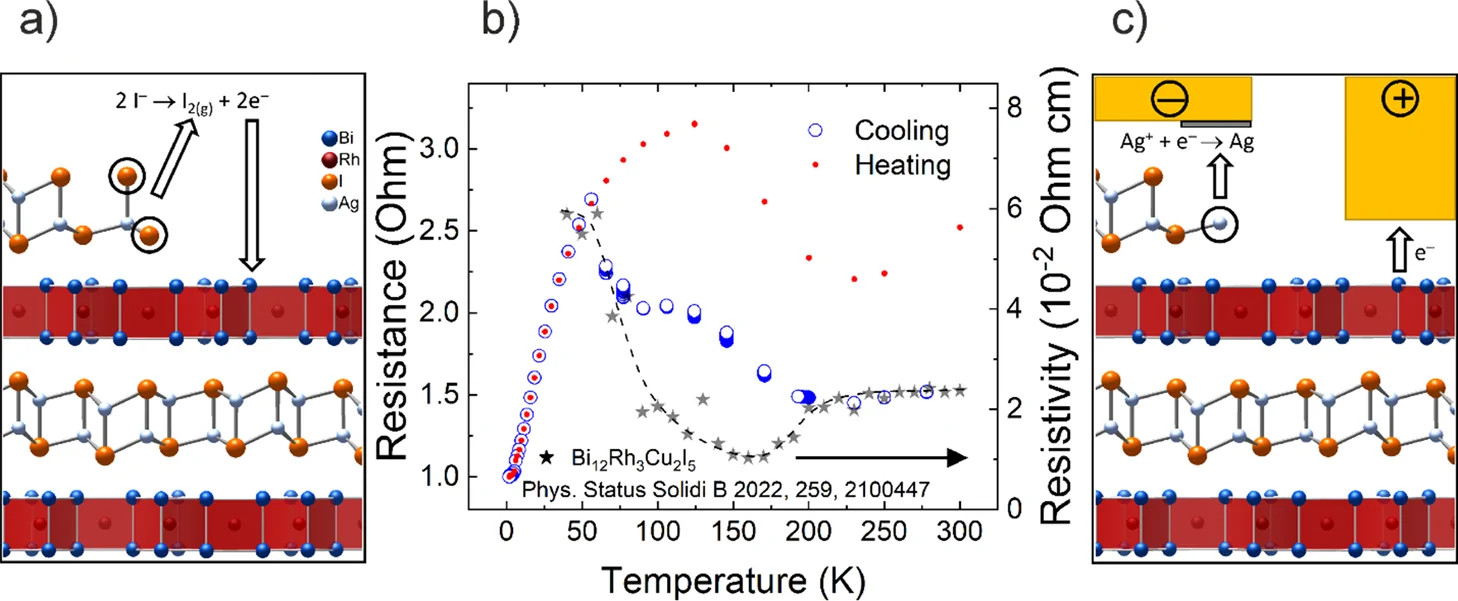
(a) Schematic illustration of iodine loss from the spacer layer terminating the crystal’s main faces (001), resulting in intrinsic *n*-doping of the pristine crystal. (b) Resistance of a Bi_12_Rh_3_Ag_6_I_9_ crystal measured along the *ab* plane as a function of temperature (and time). Above 50 K, the heating and cooling curves differ markedly. During the first cooling cycle, the resistance shows a slow time-dependent drift at temperatures above 50 K, as revealed by repeated measurements at constant temperature, consistent with a continuous electrochemical process. Data from a four-point STM measurement on Bi_12_Rh_3_Cu_2_I_5_ from Ref. (13) are included for comparison. (c) Schematic illustration of current-induced electrochemical processes causing silver loss and thereby reducing the n-type doping of the material.

**Figure 8:**
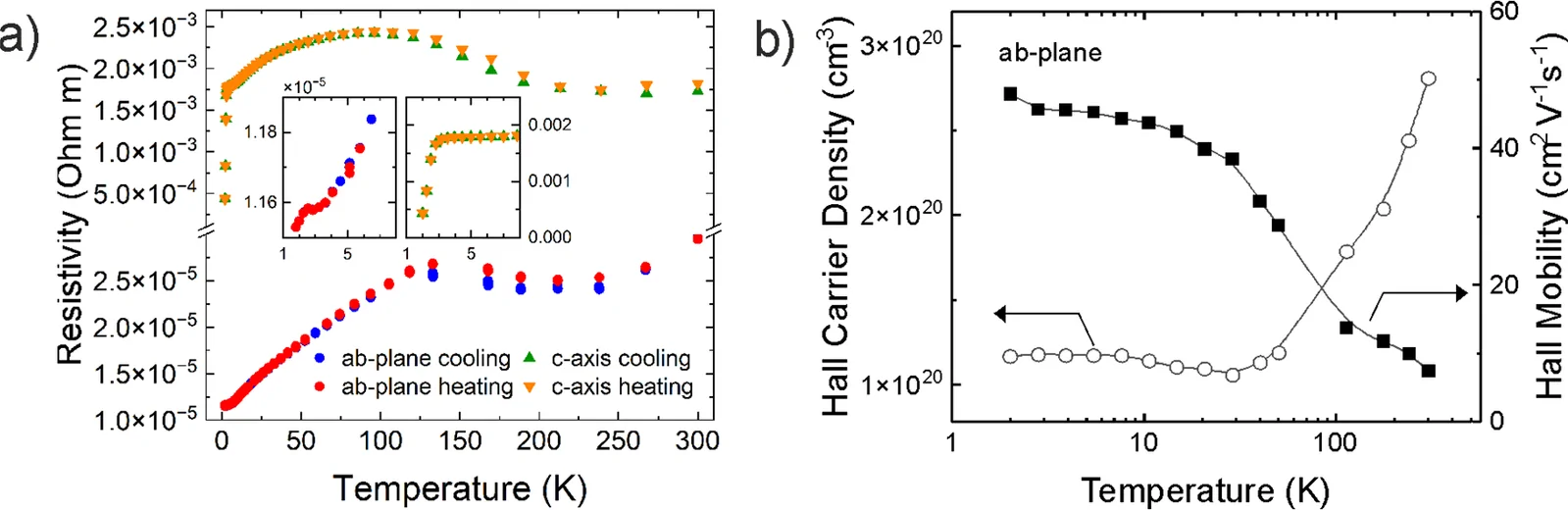
(a) Temperature-dependence of the electrical resistivity of a pretreated, substantially de-doped Bi_12_Rh_3_Ag_6_I_9_ crystal measured in four-point configuration parallel to the *ab*-plane and parallel to the *c*-axis (stacking direction). The insets provide a magnified view of the resistivity below 8 K. (b) Hall carrier density and mobility as a function of temperature parallel to the *ab*-plane of a pristine crystal.

A plausible origin of the initially enhanced conductivity is intrinsic *n*-doping caused by iodine loss from the [Ag_6_I_8_]^2–^ spacer at the crystal’s main faces (Figure 7a), a termination effect that may be further promoted by vacuum exposure, for example during ARPES measurements. The released electrons are expected to populate states in the conduction band associated mainly with the adjacent intermetallic layers in the near-surface region. The influence of the applied current is directly evident in the cooling data: at stabilized temperatures above 50 K, the resistance increases steadily during continuous measurement over 30 min, consistent with an ongoing in-operando electrochemical process. Below 50 K, presumably because ionic motion becomes too slow on the experimental time scale, this time-dependent drift becomes negligible. After the first temperature-current cycle, the heating and cooling curves nearly coincide over the entire temperature range (Figure 8a). These observations indicate that the initial excess electron density can be reduced during measurement.

As illustrated schematically in Figure 7c, the applied bias can drive migration of mobile Ag^+^ through the crystal, followed by reduction and deposition at the cathodic side, while charge compensation at the opposite side removes electrons from conduction-band states of the intermetallic layers. In this way, the initial n-type doping of the pristine crystal is reduced, giving rise to the higher resistance observed on reheating. The fact that this effect is largely absent in subsequent cycles indicates a self-limiting process rather than progressive overoxidation. A plausible explanation is that removal of electrons from antibonding conduction-band states is energetically accessible, whereas further oxidation would require depletion of bonding valence-band states across the band gap and is therefore not expected under the moderate voltages applied here. Overall, the two-point measurements suggest that Bi_12_Rh_3_Ag_6_I_9_ can be driven from an initially strongly n-doped state toward a state of markedly lower electron doping and substantially reduced bulk conduction. Surface-related electrochemical processes of this kind are also likely to contribute significantly to the apparent carrier concentration extracted from Hall measurements on pristine crystals.

To clarify the relation between the two-point and the following four-point measurements, normalized resistance data are compared in the Supporting Information (Figure S11). The two-point measurement was performed on a pristine crystal and therefore captures the first cycle, including the constant-temperature drift shown in Figure 7b. The four-point measurement was performed on a separate crystal from the same batch after mechanical trimming to defined geometry, temperature and field cycling, current flow, and vacuum exposure. Accordingly, the first cooling branch of the pristine two-point crystal and the cooling branch of the pretreated four-point crystal are not expected to be identical. After the first cycle, however, the normalized heating branch of the two-point measurement shows the same characteristic temperature dependence as the four-point data, including the broad maximum near 130 K (Figure S11). Thus, the characteristic transport behavior of the conditioned material is reproduced in a contact-independent geometry.

To quantify the transport anisotropy after this initial electrochemical conditioning, resistivity was measured in four-point configuration using a low-frequency lock-in technique on oriented crystals of Bi_12_Rh_3_Ag_6_I_9_ trimmed to defined shapes. Representative data obtained during a second cooling cycle, that is, after a first temperature-current cycle had already reduced the initial n-doping, are shown in Figure 8a. The resistivity along the *c* axis exceeds that within the *ab* plane by about two orders of magnitude, consistent with comparatively efficient charge transport within the intermetallic layers and much more strongly impeded transport across the salt-like spacer layers. In both directions, the resistivity shows a broad maximum near 130 K, in agreement with the characteristic temperature identified in the two-point measurement after the initial cycle. Some residual irreversibility remains above 130 K, but it is much weaker than in the pristine crystal. Below 130 K, the resistivity decreases upon cooling in both directions rather than showing the thermally activated increase expected for a simple semiconductor. Within the *ab* plane, this behavior is consistent with transport dominated by a low-dimensional electronic subsystem whose mean free path increases on cooling. Along the *c* axis, the decrease is weaker but still pronounced. Particularly notable is the steep drop in ρ_c_ below about 3.2 K, accompanied by a much smaller feature in ρ_ab_ between 3.2 and 1.8 K (Figure 8a, inset).

The charge carrier concentration and mobility were determined along the *ab* plane by Hall-effect measurements on a pristine crystal (Figure 8b). In agreement with the ARPES results, the material is clearly n-doped. Upon cooling from 300 to 40 K, the apparent carrier concentration decreases from about 3 × 10^20^ cm^–3^ to 1 × 10^20^ cm^–3^ and then remains nearly constant below 50 K, which coincides with the temperature range in which ionic motion, and therefore electrochemical de-doping, becomes very slow. Together with the spectroscopic evidence, these values are consistent with a narrow-gap degenerate semiconductor. However, because bulk, near-surface, and possibly other parallel conduction channels may coexist, the extracted carrier concentration and mobility should be regarded as effective Hall parameters rather than intrinsic bulk quantities. The mobility within the *ab* plane lies in the range of ordinary metals, increasing from about 10 cm^2^ V^–1^ s^–1^ at room temperature to about 48 cm^2^ V^–1^ s^–1^ at 1.8 K, but remaining moderate overall. This comparatively moderate Hall mobility likely reflects disorder scattering and multichannel transport; if near-surface doped regions contribute appreciably to the measured conductivity, the mobility extracted from Hall and resistivity data should not be interpreted as the intrinsic drift mobility of a homogeneous bulk conductor.

Taken together, the electrical transport data indicate that several conduction channels coexist in Bi_12_Rh_3_Ag_6_I_9_ and that their relative contributions change during electrochemical conditioning. In the pristine state, transport likely contains both bulk and near-surface contributions and is strongly influenced by substantial electron doping. This excess electron density can be partially compensated under bias by slow electrochemical deintercalation processes involving the ion-mobile spacer. After conditioning, the resistivity remains strongly anisotropic, confirming that transport is governed predominantly by the intermetallic layers and is strongly hindered across the spacer. The decrease of both ρ_ab_ and ρ_c_ below about 130 K shows that the transport of the conditioned crystal is no longer dominated by simple activated conduction of the semiconducting bulk. Thus, electrochemical conditioning suppresses the initially dominant trivial conduction channels and increases the relative importance of low-dimensional boundary-related transport. In view of the weak-topological classification of Bi_12_Rh_3_Ag_6_I_9_ and the close analogy to Bi_14_Rh_3_I_9_, topologically protected edge and side-facet states must therefore be considered as important contributors to the low-temperature conductance.^8,9^ A strictly quantitative separation of residual bulk, trivial surface, and topological boundary contributions is not possible from the present macroscopic Hall and resistivity data alone. The pronounced anomaly below about 3.2 K, especially for current along the stacking direction, therefore marks an unusual boundary-sensitive transport regime. Because the experiments were performed with contacting materials that become superconducting in this temperature range, coupling of superconducting electrodes to boundary-related transport channels is a plausible possibility, but its detailed physical interpretation requires dedicated investigation and will be addressed separately.

## Conclusions

Bi_12_Rh_3_Ag_6_I_9_ demonstrates that the spacer in the Bi_14_Rh_3_I_9_ family of WTIs is not a passive separator, but a chemically designable functional component. Replacing the rigid [Bi_2_I_8_]^2–^ spacer of Bi_14_Rh_3_I_9_ by an AgI-derived [Ag_6_I_8_]^2–^ layer with partially occupied tetrahedral sites introduces intrinsic two-dimensional Ag^+^ disorder and mobility, while preserving the intermetallic [(Bi_12_Rh_3_)­I]^2+^ motif that drives band inversion. The crystal structure together with DFT calculations including spin–orbit coupling provide strong evidence that Bi_12_Rh_3_Ag_6_I_9_ is a WTI, analogous to the well-established Bi_14_Rh_3_I_9_. ARPES corroborates the calculated electronic structure and yields an experimental bulk band gap of 286 meV, the widest reported for this structure family. Electrochemical impedance spectroscopy reveals a room-temperature ionic conductivity of about 10^–6^ S cm^–1^. Under modest bias, the ion-mobile spacer can act as an internal deintercalation reservoir that compensates the initial n-type doping and shifts the chemical potential toward the bulk gap. More generally, spacer chemistry—composition, defect chemistry, and ion mobility—emerges as a decisive handle to tune carrier density and surface band bending in this materials platform. This chemical control is essential for reproducibly positioning the chemical potential within the bulk band gap, thereby minimizing bulk conductivity and enabling access to the intrinsic topological edge and nontrivial side-facet response.

## Experimental Section

### Materials

Bi (≥99.9999%) and Rh (≥99.9%) were pretreated under flowing H_2_. BiI_3_ (≥99.9%) was purified by sublimation; Ag powder (200 mesh) was used as received after phase verification. All handling was performed under dry argon.

### Synthesis of Bi_12_Rh_3_Ag_6_I_9_ Powders

Bi_12_Rh_3_Ag_6_I_9_ was synthesized from mixtures of Bi, Rh, Ag, and BiI_3_ corresponding to the overall atomic ratio Bi/Rh/Ag/I = 4:1:2:3. The starting mixtures were mechanically pre-activated by ball milling, loaded into silica ampules, evacuated, and flame-sealed. Differential scanning calorimetry and ex situ annealing experiments were used to establish the formation window of the target phase. The first formation of Bi_12_Rh_3_Ag_6_I_9_ was detected in samples annealed at 265–290 °C, although these products still contained secondary phases. Phase-pure powders were reproducibly obtained after annealing for 24 h in the range 345–460 °C. At higher temperatures, Bi_12_Rh_3_Ag_6_I_9_ decomposes; DSC and ex situ experiments indicate decomposition at 479(2) °C into Bi, ht-RhBi_2_, AgI, and BiI_3_. Further details can be found in the Supporting Information.

### Crystal Growth

Platelet single crystals with lateral dimensions up to several millimeters were grown from Bi/BiI_3_-rich melts in sealed evacuated silica ampules using temperature profiles derived from the DSC and annealing studies. Typical crystals are black platelets and often show intergrowth. Details can be found in the Supporting Information.

### X-ray Diffraction

Powder X-ray diffraction was performed with Cu Kα_1_ radiation in Bragg–Brentano geometry. Single-crystal data were collected at 296 and 100 K on a Bruker Kappa APEX II CCD diffractometer (graphite(002)-monochromator, CCD-detector) using Mo Kα radiation (λ = 0.71073 Å). The structures were solved and refined in the rhombohedral space group *R*3̅*m* (hexagonal setting) using SHELXL;^23^ crystallographic details and refinement parameters are summarized in Tables S1–S4.

### Elemental Analysis

Scanning electron microscopy (SEM) was performed using a SU8020 (Hitachi) with a triple detector system for secondary and low-energy backscattered electrons (*U*_acc._ = 5 kV). The composition of selected crystals was determined by semi-quantitative energy dispersive X-ray (EDX) analysis (*U*_acc._ = 15 kV) using a Silicon Drift Detector (SDD) X-MaxN (Oxford Instruments). The data were processed (integration, pulse-pile up correction) applying the AZtec software package (Oxford Instruments, 2013). A representative EDX result is Bi/Rh/Ag/I (at.-%) = 42(2):9(2):18(2):31(2), calculated: 40:10:20:30.

### Thermal Analysis

Differential scanning calorimetry (DSC) was carried out in sealed silica ampules at heating and cooling rates of 2 K min^–1^. In the heating runs, signals at 144 and 261 °C are associated with the α/β-transition of traces of AgI and with reaction processes initiated by the melting of Bi, respectively, whereas the decomposition of Bi_12_Rh_3_Ag_6_I_9_ is observed at 479 °C. Upon cooling, a crystallization signal of Bi_12_Rh_3_Ag_6_I_9_ appears at 466 °C. For details, see Supporting Information.

### Photoelectron Spectroscopy

XPS and ARPES measurements were performed on freshly cleaved (001) crystal surfaces. XPS spectra shown in this work were recorded at PETRA III (P04); the displayed survey spectrum was obtained at *h*ν = 1200 eV and 30 K. ARPES data were recorded at 30 K with *h*ν = 260 eV after low-temperature cleavage.

### Electrochemical Impedance Spectroscopy

EIS was measured on cold-pressed pellets sintered between Ag plates. Spectra were recorded from 1 Hz to 1 MHz with a small AC excitation and analyzed by fitting equivalent-circuits to separate bulk ionic and grain-boundary contributions.

### Electronic Transport

Two-point resistance, four-point resistivity, and Hall-effect measurements were carried out on oriented single crystals with defined geometries using low-frequency lock-in techniques in a Quantum Design Dynacool cryostat. Current paths were arranged either parallel to the *ab* plane or parallel to the *c* axis. Electrical contacts were made with high-purity indium, which was cold-welded onto the crystals. For the two-point measurements, rectangular indium electrodes cut from 100 μm thick sheet material (Goodfellow) were attached to opposite sides of the crystal, contacting either the top and bottom facets or the lateral facets. Four indium wires with a diameter of 100 μm (Hauner), two on each rectangular electrode, were used for current supply and voltage sensing. Conventional four-point measurements were carried out with four individual indium wire electrodes attached directly to lateral crystal facets.

### Computational Methods

The DFT calculations were performed for an ordered periodic approximant. In this model, Ag fully occupies one set of 18 h Wyckoff positions, preserving the composition Bi_12_Rh_3_Ag_6_I_9_. For all other atoms, the calculations are based on the experimentally determined average structure. Bulk band structure and DOS calculations for Bi_12_Rh_3_Ag_6_I_9_ were performed with the Vienna Ab Initio Simulation Package (VASP),^24,25^ using the projector augmented wave (PAW) method. Local density approximation (LDA) has been used to handle exchange and correlation effects.^26^ Spin–orbit coupling (SOC) has been included self-consistently, except where explicitly stated otherwise. In the self-consistent computation, we considered 400 eV for the plane-wave cutoff, 9 × 9×3 *k*-points together with a Gaussian smearing and a smearing width of 0.005 eV. The electronic loop was considered converged below a threshold of 1 × 10^–6^ eV. For the DOS calculations we used the tetrahedron method with Blöchl corrections together with a finer *k*-grid. Calculations for charged and uncharged single layers were performed using a unit cell of 24 Å perpendicularly to the plane itself and 9 × 9×1 *k*-points to ensure to work with a well-isolated system. Concerning the analysis of the *Z*_2_ topological invariant, we started by obtaining the Hamiltonian of the intermetallic charge-compensated layer in the Wannier functions basis.^27–29^ We considered projections onto the following states: Bi p_*z*_, p_*x*_, p_*y*_; Rh d_*z*2_, d_*xz*_, d_*yz*_, d_*x*2–y2_, d_*xy*_, p_*z*_, p_*x*_, p_*y*_, s; I p_*z*_, p_*x*_, p_*y*_. Mixing between states is avoided in a large-enough energy window centered at the Fermi level, being the inclusion of these states in the optimal subspace unchanged. Finally, we computed the *Z*_2_ topological number from the Wannier charge centers for the *k*_z_ = 0.0 plane.^30,31^ The analysis of the topological properties for the whole compound was instead based on the parity eigenvalues at the TRIM points, as implemented in the FPLO code.^32^ The following software packages were used to visualize and post-process the data: VESTA,^33^ XCrySDen,^34,35^ VASPKIT.^36^

### Safety Statement

No unexpected or unusually severe hazards were encountered in this work beyond those commonly associated with high-temperature synthesis, such as evacuated silica ampules, iodine-containing reagents, high-temperature furnaces, and heavy-metal-containing compounds. Reactions in sealed ampules were carried out behind appropriate shielding, and ampules were inspected carefully before opening. Starting materials and synthesized compounds were handled using standard precautions for heavy-metal-containing inorganic solids and iodine-containing materials, including work under inert atmosphere where appropriate and disposal as heavy-metal-containing waste.

## Supplementary Material



## Notes

Synthesis optimization, DSC data, PXRD patterns, SEM/EDX analyses, crystallographic tables, and additional electronic-structure data. Further details of the crystal structure investigations may be obtained from the joint CCDC/FIZ Karlsruhe online deposition service: https://www.ccdc.cam.ac.uk/structures/by quoting the deposition numbers CSD-2220351 (296 K) and CSD-2290579 (100 K) (PDF)
