# Supplementary material for: Bi12Rh3Ag6I9: Mobile Silver Ions and Surface-Dominated Transport in a Layered Weak Topological Insulator
Source: Inorg Chem. 2026 Oct 6;65(41):24145–54. doi: 10.1021/acs.inorgchem.6c02517 (PMC13685412; doi:10.1021/acs.inorgchem.6c02517)
Supplement: Supplementary file 1 [file ic6c02517_si_001.pdf]

Supporting Information

$Bi_{12}Rh_3Ag_6I_9$: Mobile Silver Ions and Surface-Dominated Transport in a Layered Weak Topological Insulator

*Eduardo Carrillo-Aravena,[1,2] Johannes Heßdörfer,[2,3] Christian N. Saggau,[4,5,6] Armando Consiglio,[2,3] Shailja Sharma,[4] Friedrich Reinert,[2,3] Giorgio Sangiovanni,[2,3] Domenico Di Sante,[7,8] Oleg Janson,[4] Kornelius Nielsch,[4,9,10] Nicolás Pérez,[4] Michael Ruck[1,2]**

[1] *Faculty of Chemistry and Food Chemistry, Dresden University of Technology, 01062 Dresden, Germany.*

[2] *Würzburg-Dresden Cluster of Excellence ctd.qmat, Dresden University of Technology, 01062 Dresden, Germany.*

[3] *Faculty of Physics and Astronomy, University of Würzburg, 97074 Würzburg, Germany.*

[4] *Leibniz Institute for Solid State and Materials Research, 01069 Dresden, Germany.*

[5] *Department of Electrical and Photonics Engineering, Technical University of Denmark, 2800 Kgs. Lyngby, Denmark.*

[6] *Center for Silicon Photonics for Optical Communications (SPOC), Technical University of Denmark, 2800 Kgs. Lyngby, Denmark.*

[7] *Department of Physics and Astronomy, University of Bologna, 40127 Bologna, Italy.*

[8] *Center for Computational Quantum Physics, Flatiron Institute, New York, NY 10010, USA.*

[9] *Institute of Applied Physics, Dresden University of Technology, 01062 Dresden, Germany.*

[10] *Institute of Materials Science, Dresden University of Technology, 01062 Dresden, Germany.*

*Corresponding Author E-mail: michael.ruck@tu-dresden.de

Contents

S1. Compounds of the $Bi_{14}Rh_3I_9$ family S4

Figure S1. Layered compounds of the $Bi_{14}Rh_3I_9$ family are periodic heterostructures of (a) the 2D-TI $[(Bi_{12}Rh_3)I]^{2+}$ and a topologically trivial spacer such as (b) the three-atoms-thick layer $[Sn_3I_8]^{2-}$ in $Bi_{12}Rh_3Sn_3I_9$ or (c) the one-atom-thick layer $[Cu_2I_4]^{2-}$ in $Bi_{12}Rh_3Cu_2I_5$........ S4

S2. Additional Information on synthesis optimization and crystal growth S4

Figure S2. SEM images of $Bi_{12}Rh_3Ag_6I_9$ crystals. (a, b) Typical morphology and intergrowth. (c) Areas used for determining the composition by EDX analysis. (d) Elemental maps for Bi, Rh, Ag, and I show homogeneous distributions over the main crystal area within the spatial resolution of SEM–EDX. A small AgI particle, visible at the surface and edge of the specimen, is not representative of the main crystal body. (e) Representative EDX spectra give compositions close to the expected Bi:Rh:Ag:I ratio of 40:10:20:30; minor deviations, especially slightly lower local Ag contents, are consistent with the known tendency toward intergrowth or local spacer variation. S5

Figure S3. DSC measurement of a mixture of Bi, Rh, Ag and BiI_3 with the molar ratio 3:1:2:1 heated from 100 °C to 720 °C and cooled at 2 K min^{-1}. S6

Figure S4. Powder diffractogram (Cu-$K\alpha_1$) of three ball-milled mixtures of Bi-Rh-Ag-BiI_3 (molar ratio Bi:Rh:Ag:I = 4:1:2:3) ramped at 2 K min^{-1} from room temperature to 265 °C and subsequently annealed for 0, 5 or 10 min and quenched to RT. Calculated diffraction patterns are given as reference for products and by-products. S6

Figure S5. Powder diffractogram (Cu-$K\alpha_1$) of a ball-milled mixture of Bi-Rh-Ag-BiI_3 (molar ratio Bi:Rh:Ag:I = 4:1:2:3) ramped at 2 K min^{-1} from room temperature to 265 °C and subsequently annealed for 24 h and quenched to RT. S7

Figure S6. Powder diffractogram (Cu-$K\alpha_1$) of a ball-milled mixture of Bi-Rh-Ag-BiI_3 (molar ratio Bi:Rh:Ag:I = 4:1:2:3) ramped at 2 K min^{-1} from room temperature to 290 °C and subsequently annealed for 24 h and quenched to RT. The major relative phases are the quaternary layered phases $Bi_{12}Rh_3Ag_2I_5$ and the target compound $Bi_{12}Rh_3Ag_6I_9$. S7

Figure S7. Top: Powder diffractogram (Cu-$K\alpha_1$) of three ball-milled samples of a Bi-Rh-Ag-BiI_3–mixture (molar ratio Bi:Rh:Ag:I = 4:1:2:3) ramped at 2 K min^{-1} from room temperature to 345, 400 or 460 °C and subsequently annealed for 24 h and quenched to RT. The target compound $Bi_{12}Rh_3Ag_6I_9$ is the only phase observed. Below: A Le Bail and profile fit of one of the diffractograms of $Bi_{12}Rh_3Ag_6I_9$. The observed reflections are accounted for by $Bi_{12}Rh_3Ag_6I_9$, and no significant additional reflections attributable to secondary crystalline phases are observed. The high absorption, the platelet morphology and associated preferred orientation, and diffuse scattering due to stacking disorder strongly affect the intensities. S8

Figure S8. Powder diffractogram of a ball-milled mixture of Bi-Rh-Ag-BiI_3 (molar ratio Bi : Rh : Ag : I = 4 : 1 : 2 : 3) ramped at 2 K min^{-1} from room temperature to 500 °C and subsequently annealed for 24 h and quenched to RT. $Bi_{12}Rh_3Ag_6I_9$ decomposes mainly into ht-$RhBi_2$, AgI and Bi (and BiI_3, which condensed at the other end of the ampule that was quenched first). S9

S3. Crystal structure S10

Table S1. Crystal data and structure refinement for $Bi_{12}Rh_3Ag_6I_9$ at 296 and 100 K. S10

Table S2. Atomic coordinates and U_{eq} [pm^2] for $Bi_{12}Rh_3Ag_6I_9$ at 296 K. U_{eq} is defined as 1/3 of the trace of the orthogonalized U_{ij} tensor. The occupancies of Ag1 and Ag2 are 0.75. .... S11

Table S3. Atomic coordinates and U_{eq} [pm^2] for $Bi_{12}Rh_3Ag_6I_9$ at 100 K. The occupancies are 0.69 for Ag1 and 0.93 for Ag2. .... S11

Table S4. Selected interatomic distances [Å] in $Bi_{12}Rh_3Ag_6I_9$ at 296 K and 100 K. Symmetry codes: #1: 1–x+y, 1–x, z; #2: –3+y, –5–x+y, –z; #3: 1–y, +x–y, +z; #4: –x+y, –x, +z. S11

Figure S9. Comparison between a section of β-AgI (a,c) and the spacer in $Bi_{12}Rh_3Ag_6I_9$ (b, d). The latter has a 75% statistical occupancy of the tetrahedral voids. The similarity between the two structures is easily seen from the top view (a,b). In the spacer layer of $Bi_{12}Rh_3Ag_6I_9$ additional tetrahedral voids are filled, which can alternatively be regarded as a rearrangement of Ag^+ cations from neighboring sites. Arrows highlight the most obvious differences in atomic positions. In γ-AgI, the same layers as in β-AgI are found perpendicular to the <111> diagonals. S12

S4. Electronic structure **S13**

Figure S10. a) Charge density isosurface for $Bi_{12}Rh_3Ag_6I_9$. Note the weak interaction among spacer and intermetallic layers. The colors used for the isosurface are not related to the colors of next panels; b-d) Orbital and element-projected band structures along Γ-A path. *d* orbitals are represented by dark-red points, *p* orbitals by orange points and *s* orbitals by yellow ones. S13

Table S5. Structure parameters for the DFT calculations on $Bi_{12}Rh_3Ag_6I_9$. S13

Figure S11. Comparison of normalized resistance data obtained from a two-point measurement on a pristine $Bi_{12}Rh_3Ag_6I_9$ crystal and from a four-point measurement on a pretreated crystal from the same batch. The same contacting material and attachment method were used in both cases. The two-point measurement captures the first temperature and current cycle of a pristine crystal, whereas the four-point measurement was performed after vacuum exposure, current flow, temperature and field cycling, and mechanical trimming to defined geometry. The cooling branches (left) therefore compare different initial states. The heating branches (right) compare conditioned states and show the same characteristic temperature dependence, including the broad maximum near 130 K. The remaining differences mainly reflect different sample geometries, contact configurations, and pretreatment histories. S15

S1. Compounds of the $Bi_{14}Rh_3I_9$ family

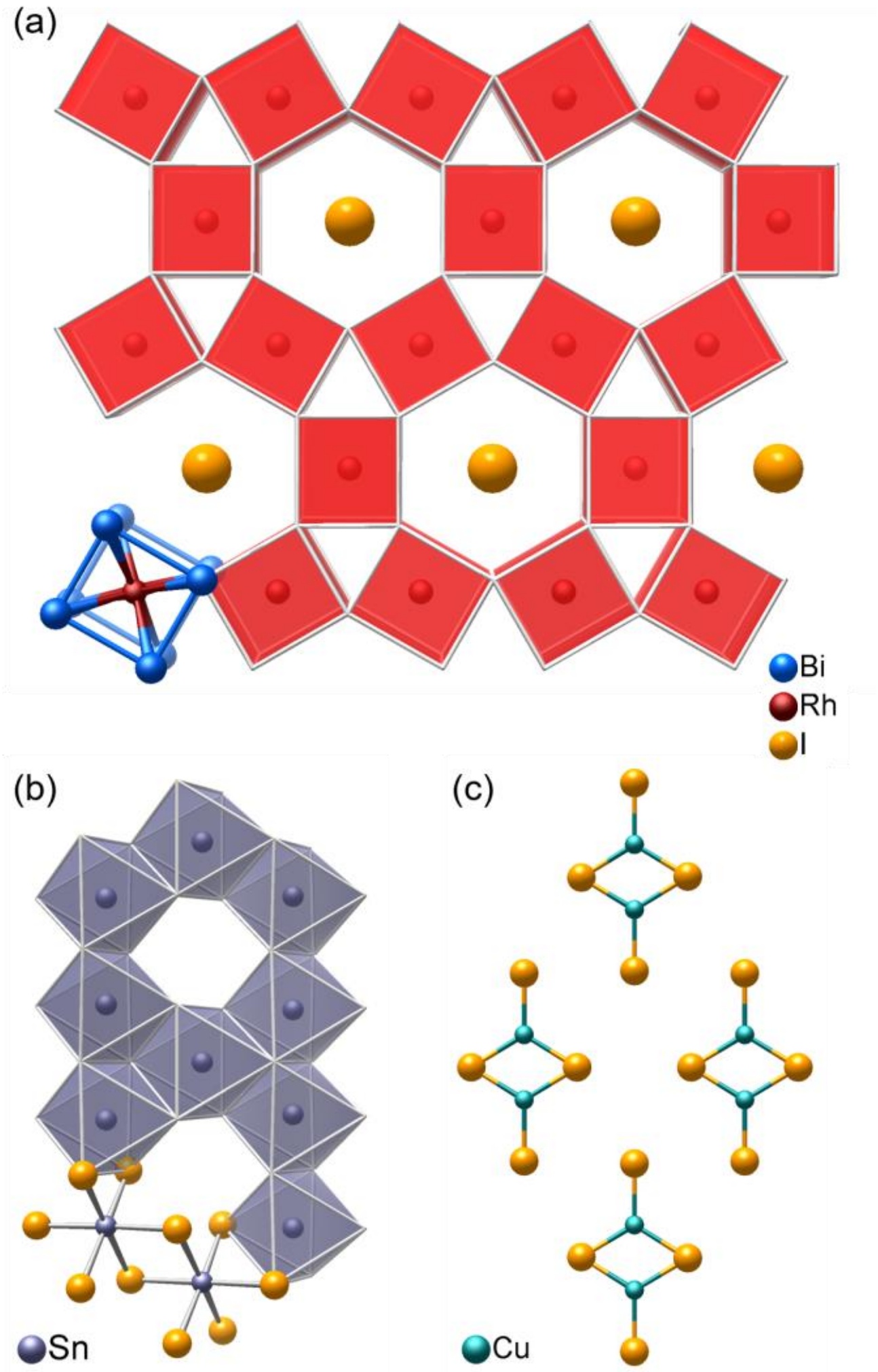

Figure S1. Layered compounds of the $Bi_{14}Rh_3I_9$ family are periodic heterostructures of (a) the 2D-TI $[(Bi_{12}Rh_3)I]^{2+}$ and a topologically trivial spacer such as (b) the three-atoms-thick layer $[Sn_3I_8]^{2-}$ in $Bi_{12}Rh_3Sn_3I_9$ or (c) the one-atom-thick layer $[Cu_2I_4]^{2-}$ in $Bi_{12}Rh_3Cu_2I_5$.

S2. Additional Information on synthesis optimization and crystal growth

Detailed DSC, ex situ phase-analysis, PXRD, SEM, and EDX data supporting the synthesis and crystal-growth procedures summarized in the main text are compiled in Figures S2–S8. These data show that $Bi_{12}Rh_3Ag_6I_9$ first forms once the starting mixture is heated above 261 °C, but its formation is kinetically hindered. The phase is already detectable after annealing at 265–290 °C, whereas phase-pure powders are obtained reproducibly only after annealing for 24 h in the range 345–460 °C. At higher temperatures, $Bi_{12}Rh_3Ag_6I_9$ decomposes near 479 °C.

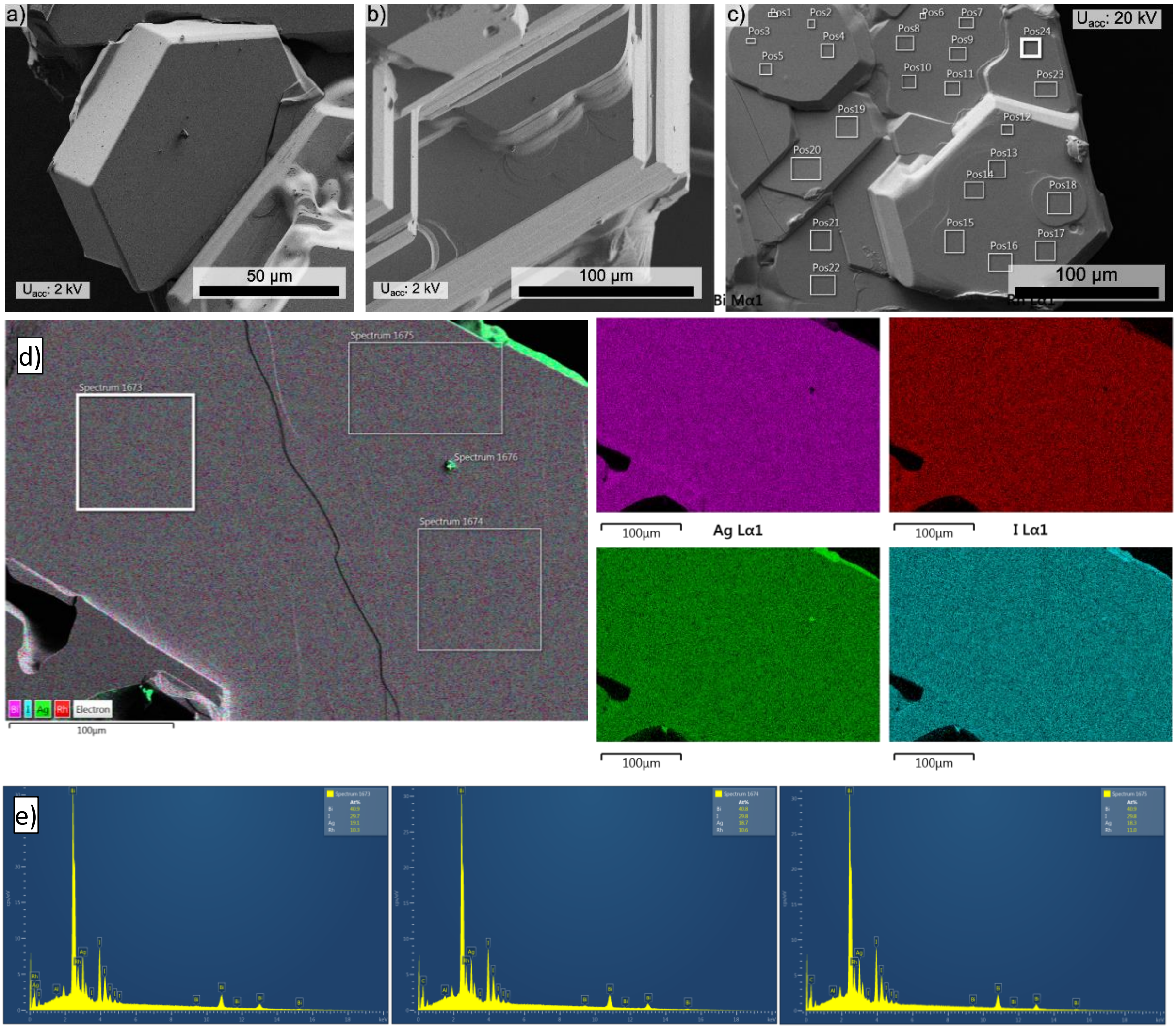

Figure S2. SEM images of $Bi_{12}Rh_3Ag_6I_9$ crystals. (a, b) Typical morphology and intergrowth. (c) Areas used for determining the composition by EDX analysis. (d) Elemental maps for Bi, Rh, Ag, and I show homogeneous distributions over the main crystal area within the spatial resolution of SEM–EDX. A small AgI particle, visible at the surface and edge of the specimen, is not representative of the main crystal body. (e) Representative EDX spectra give compositions close to the expected Bi:Rh:Ag:I ratio of 40:10:20:30; minor deviations, especially slightly lower local Ag contents, are consistent with the known tendency toward intergrowth or local spacer variation.

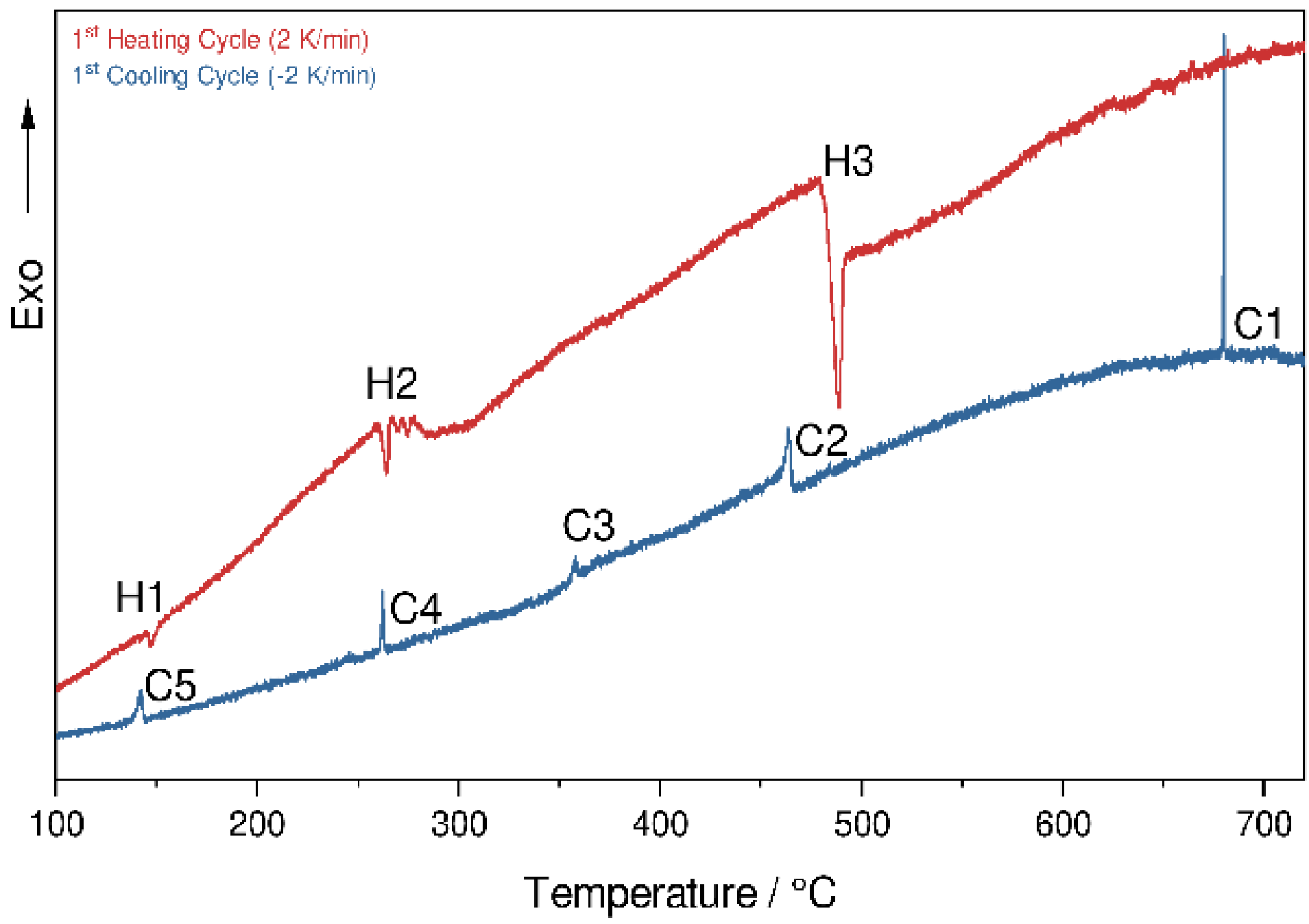

Figure S3. DSC measurement of a mixture of Bi, Rh, Ag and BiI_3 with the molar ratio 3:1:2:1 heated from 100 °C to 720 °C and cooled at 2 K min^{-1}.

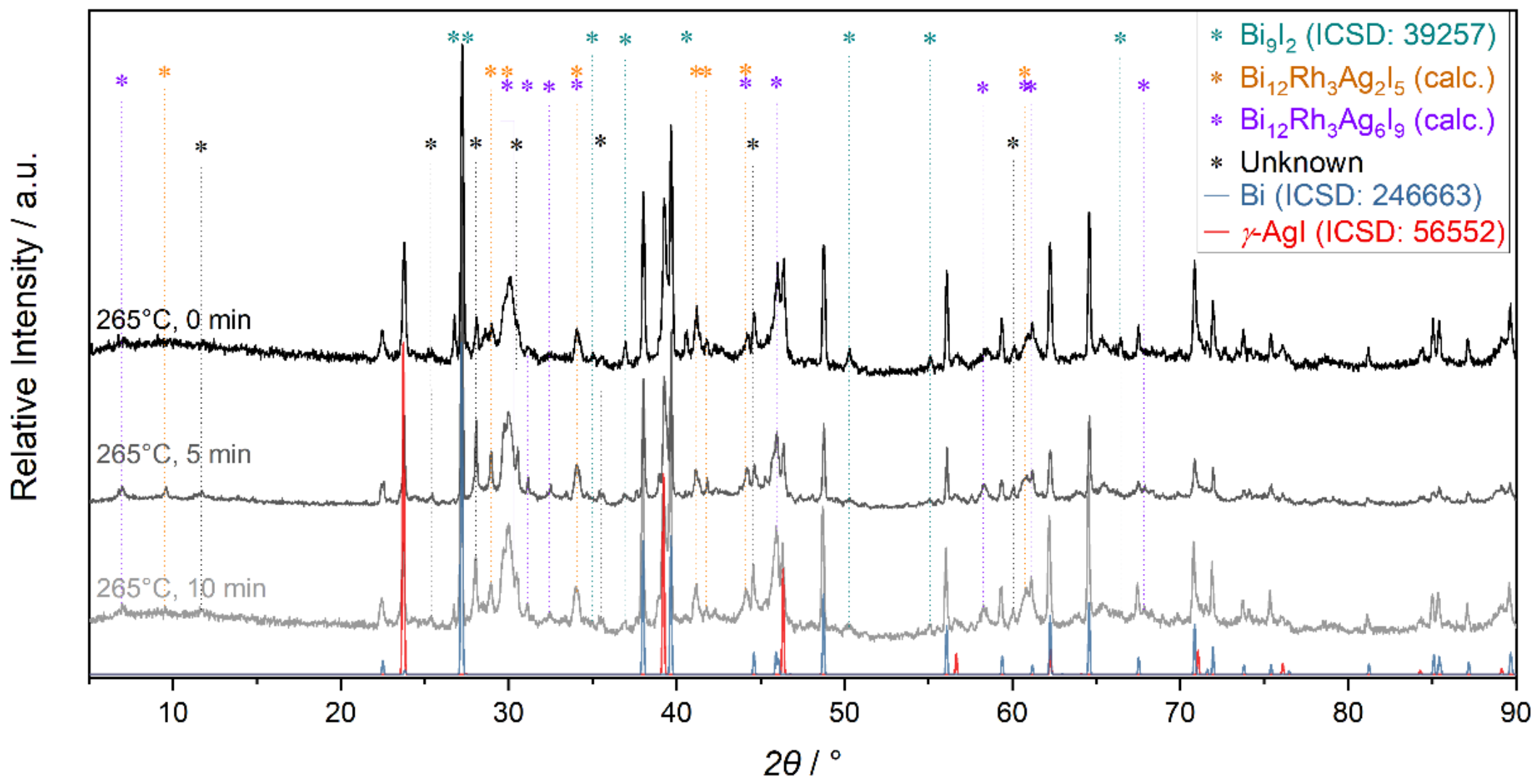

Figure S4. Powder diffractogram (Cu-Kα_1) of three ball-milled mixtures of Bi-Rh-Ag-BiI_3 (molar ratio Bi:Rh:Ag:I = 4:1:2:3) ramped at 2 K min^{-1} from room temperature to 265 °C and subsequently annealed for 0, 5 or 10 min and quenched to RT. Calculated diffraction patterns are given as reference for products and by-products.

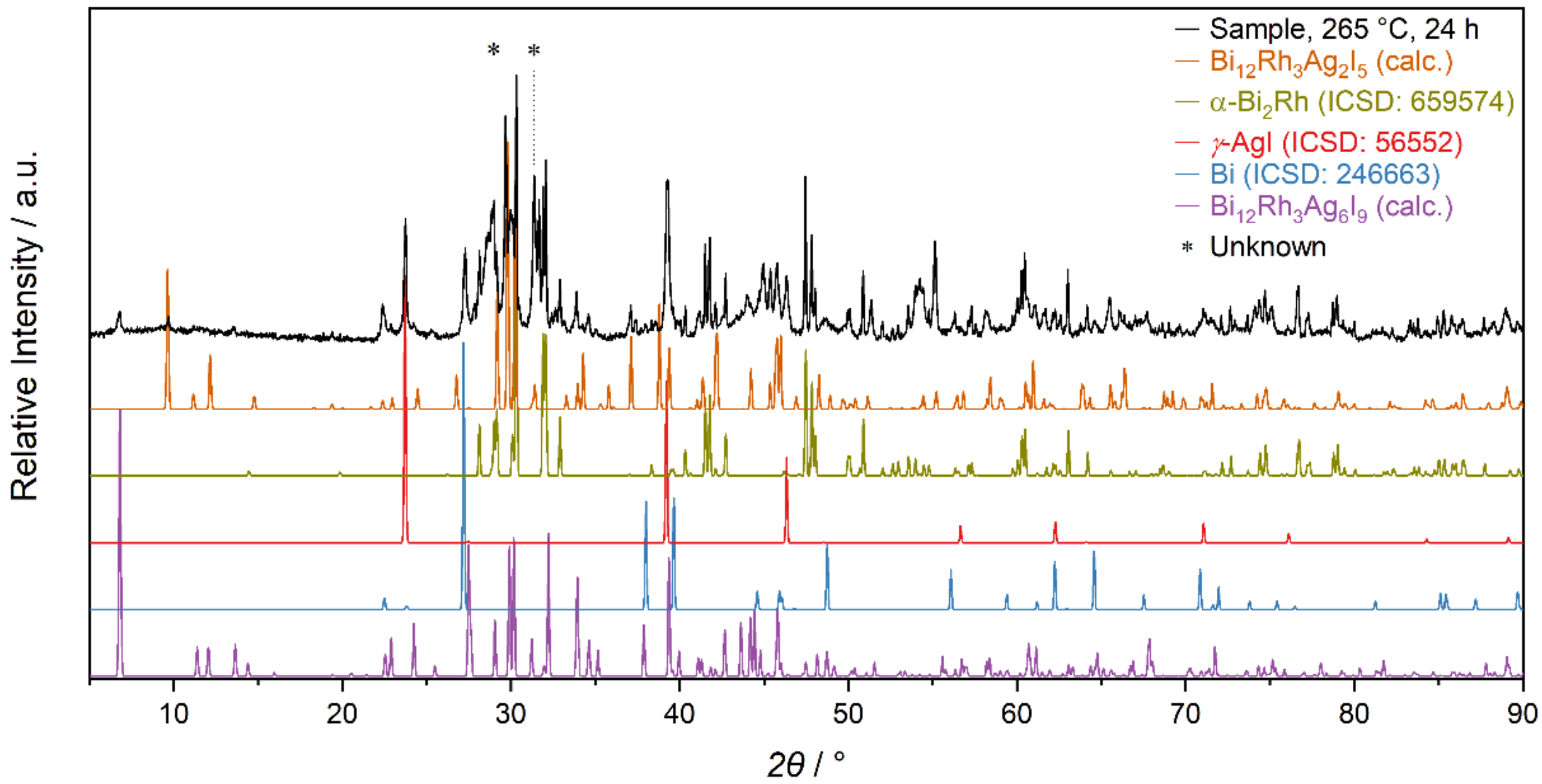

Figure S5. Powder diffractogram (Cu-Kα_1) of a ball-milled mixture of Bi-Rh-Ag-BiI_3 (molar ratio Bi:Rh:Ag:I = 4:1:2:3) ramped at 2 K min^{-1} from room temperature to 265 °C and subsequently annealed for 24 h and quenched to RT.

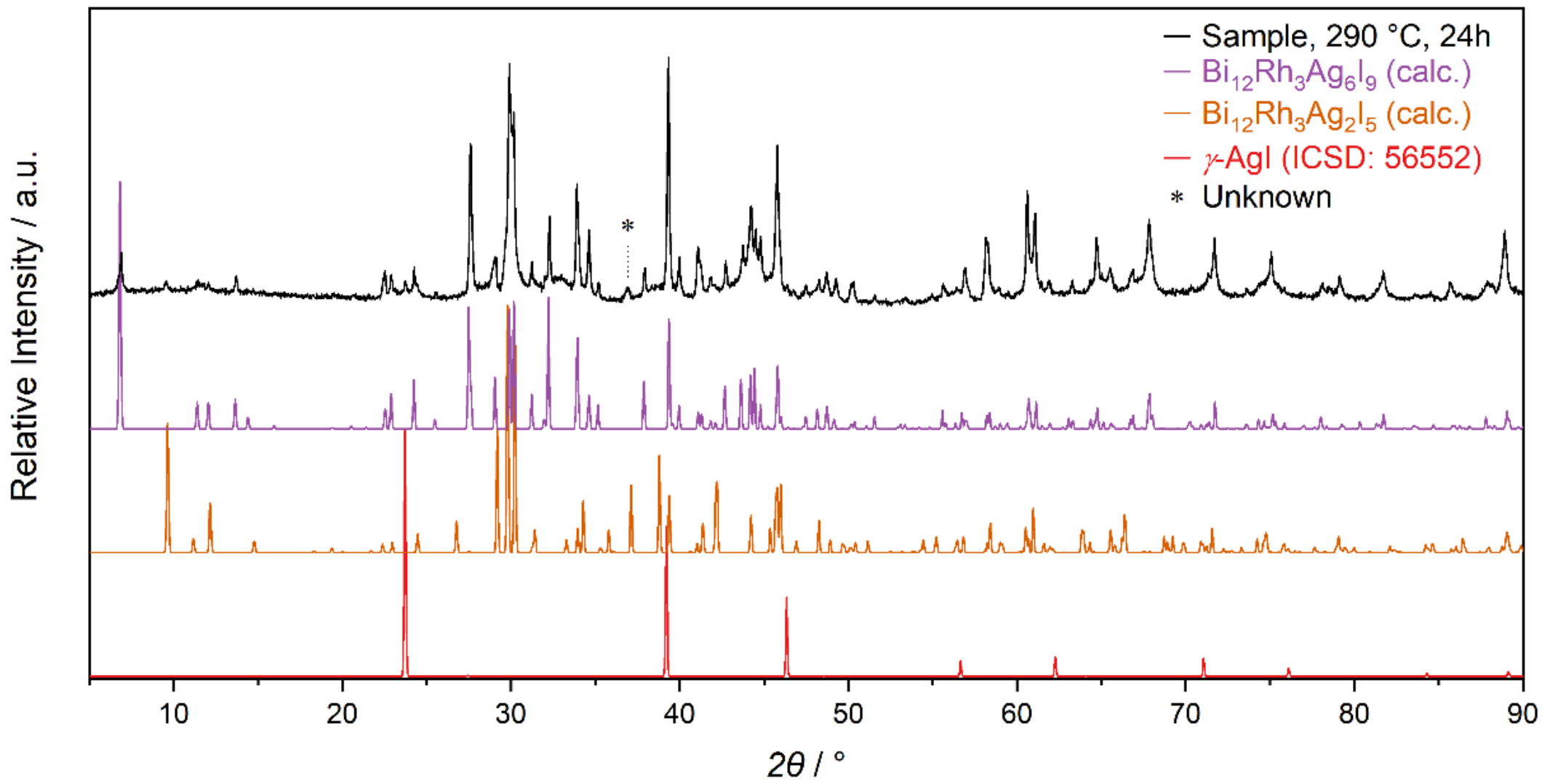

Figure S6. Powder diffractogram (Cu-Kα_1) of a ball-milled mixture of Bi-Rh-Ag-BiI_3 (molar ratio Bi:Rh:Ag:I = 4:1:2:3) ramped at 2 K min^{-1} from room temperature to 290 °C and subsequently annealed for 24 h and quenched to RT. The major relative phases are the quaternary layered phases $Bi_{12}Rh_3Ag_2I_5$ and the target compound $Bi_{12}Rh_3Ag_6I_9$.

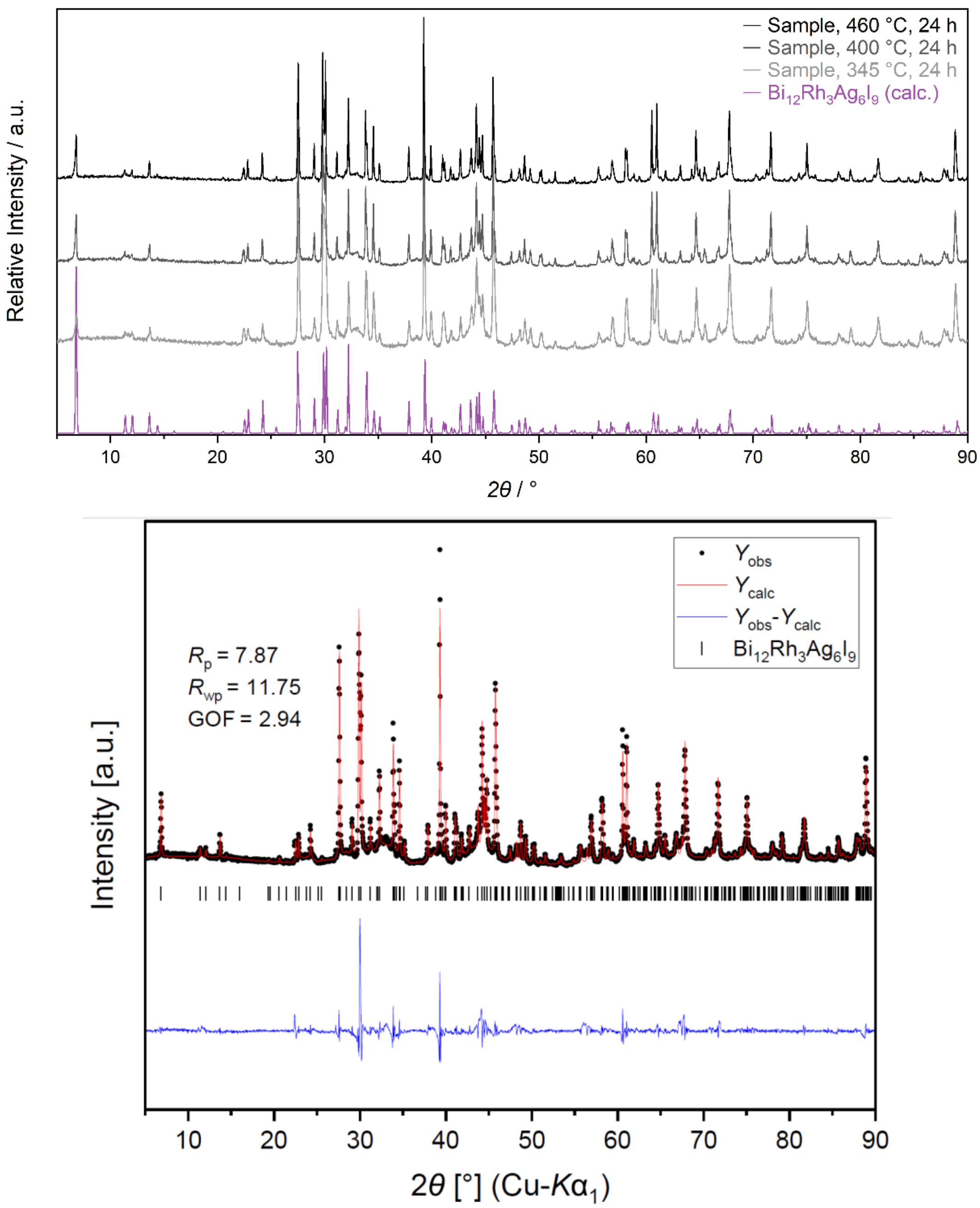

Figure S7. Top: Powder diffractogram (Cu-Kα_1) of three ball-milled samples of a Bi-Rh-Ag-BiI_3–mixture (molar ratio Bi:Rh:Ag:I = 4:1:2:3) ramped at 2 K min^{-1} from room temperature to 345, 400 or 460 °C and subsequently annealed for 24 h and quenched to RT. The target compound $Bi_{12}Rh_3Ag_6I_9$ is the only phase observed. Below: A Le Bail and profile fit of one of the powder diffractograms of $Bi_{12}Rh_3Ag_6I_9$. The observed reflections are accounted for by $Bi_{12}Rh_3Ag_6I_9$, and no significant additional reflections attributable to secondary crystalline phases are observed. The high absorption, the platelet morphology and associated preferred orientation, and diffuse scattering due to stacking disorder strongly affect the intensities.

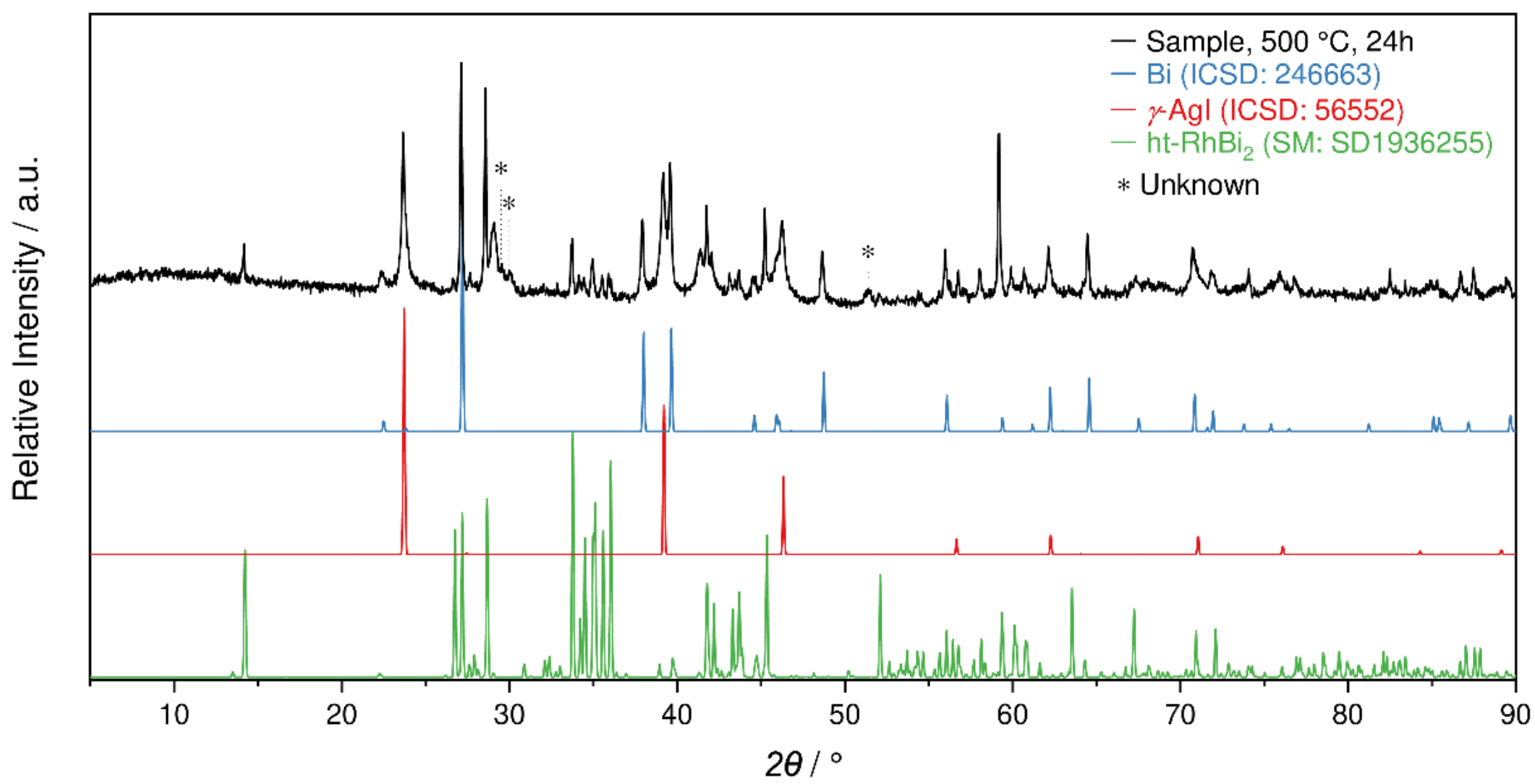

Figure S8. Powder diffractogram of a ball-milled mixture of Bi-Rh-Ag-BiI_3 (molar ratio Bi : Rh : Ag : I = 4 : 1 : 2 : 3) ramped at 2 K min^{-1} from room temperature to 500 °C and subsequently annealed for 24 h and quenched to RT. $Bi_{12}Rh_3Ag_6I_9$ decomposes mainly into ht-$RhBi_2$, AgI and Bi (and BiI_3, which condensed at the other end of the ampule that was quenched first).

S3. Crystal structure

Table S1. Crystal data and structure refinement for $Bi_{12}Rh_3Ag_6I_9$ at 296 and 100 K.

Empirical formula	$Bi_{12}Rh_3Ag_6I_9$	
CSD number	2220351	2290579
Temperature [K]	296(2)	100(2)
Formula weight	4605.81	
Crystal system, Z	trigonal, 3	
Space group (number)	$R\bar{3}m$ (166)	
a [Å]	9.1585(3)	9.1272(2)
b [Å]	9.1585(3)	9.1272(2)
c [Å]	38.9458(14)	38.6330(7)
V [Å^3]	2829.0(2)	2787.2(2)
ρ_{calc} [g cm^{-3}]	8.110	8.232
Diffractometer	Bruker Kappa APEX II CCD, graphite (002) monochromator	
μ [mm^{-1}]	67.411	68.424
F(000)	5670	
Crystal size [mm^3]	0.100 × 0.040 × 0.014	
Crystal color	black	
Crystal shape	platelet	
Radiation	MoK_α (λ = 0.71073 Å)	
2θ range [°]	3.14 to 66.48	5.26 to 72.80
Resolution [Å]	0.65	0.60
Index ranges	$-14 \le h \le 14$<br>$-14 \le k \le 14$<br>$-60 \le l \le 59$	$-15 \le h \le 15$<br>$-15 \le k \le 15$<br>$-64 \le l \le 64$
Reflections measured	33811	112956
Independent reflections	1416	1741
R_{int}	0.0607	0.0614
R_σ	0.0221	0.0135
Data / Restraints / Parameters	1416 / 0 / 38	1741 / 0 / 38
Goodness-of-fit on F^2	1.063	1.154
Final R indexes [$I \ge 2\sigma(I)$]	R_1 = 0.0287<br>wR_2 = 0.0726	R_1 = 0.0175<br>wR_2 = 0.0454
Final R indexes [all data]	R_1 = 0.0333<br>wR_2 = 0.0746	R_1 = 0.0181<br>wR_2 = 0.0457
Largest peak/hole [eÅ^{-3}]	5.12/–4.42[a]	4.39/–2.34[a]
Extinction coefficient	22.8(12)·10^{-5}	5.7(5)·10^{-5}

[a] The crystal described here, as well as many other investigated crystals, consisted predominantly of $Bi_{12}Rh_3Ag_6I_9$ with the three-atom-thick $[Ag_6I_8]^{2-}$ spacer. Occasional insertion of a one-atom-thick $[Ag_2I_4]^{2-}$ spacer, corresponding to the closely related compound $Bi_{12}Rh_3Ag_2I_5$, gives rise to comparatively high residual electron density maxima.

Table S2. Atomic coordinates and U_{eq} [pm^2] for $Bi_{12}Rh_3Ag_6I_9$ at 296 K. U_{eq} is defined as 1/3 of the trace of the orthogonalized U_{ij} tensor. The occupancies of Ag1 and Ag2 are 0.75.

Atom	Wyckoff pos.	*x*	*y*	*z*	U_{eq}
Bi1	18*h*	0.43459(4)	0.21729(2)	0.04082(2)	166(1)
Bi2	18*h*	0.21737(2)	0.43475(4)	0.04058(2)	166(1)
Rh	9*e*	0	0	0	118(2)
I1	3*a*	0	0	0	247(3)
I2	6c	0	0	0.10801(4)	399(3)
I3	18*h*	0.16233(6)	0.3247(1)	0.21279(2)	385(2)
Ag1	18*h*	0.1610(1)	0.3220(3)	0.13772(6)	679(5)
Ag2	6*c*	0	0	0.18129(9)	646(8)

Table S3. Atomic coordinates and U_{eq} [pm^2] for $Bi_{12}Rh_3Ag_6I_9$ at 100 K. The occupancies are 0.69 for Ag1 and 0.93 for Ag2.

Atom	Wyckoff pos.	*x*	*y*	*z*	U_{eq}
Bi1	18*h*	0.43444(2)	0.21722(2)	0.04107(2)	54.7(5)
Bi2	18*h*	0.21726(2)	0.43452(2)	0.04076(2)	54.2(5)
Rh	9*e*	0	0	0	40.2(8)
I1	3*a*	0	0	0	71(1)
I2	6c	0	0	0.10787(2)	141(1)
I3	18*h*	0.16370(2)	0.32739(5)	0.21294(2)	139.8(7)
Ag1	18*h*	0.16091(5)	0.3218(1)	0.13789(2)	202(1)
Ag2	6*c*	0	0	0.18128(2)	156(1)

Table S4. Selected interatomic distances [Å] in $Bi_{12}Rh_3Ag_6I_9$ at 296 K and 100 K. Symmetry codes: #1: 1–x+y, 1–x, z; #2: –3+y, –5–x+y, –z; #3: 1–y, +x–y, +z; #4: –x+y, –x, +z.

Atom pairs	Distance (296 K)	Distance (100 K)
Bi1–Rh1	2.8353(2)	2.8272(1)
Bi1–Bi2$^{\#2}$	3.1702(5)	3.1616(2)
Bi1–Bi1$^{\#3}$	3.1882(6)	3.1794(3)
Bi1–Bi1$^{\#1}$	3.1883(6)	3.1794(3)
Bi1–Bi2	3.4476(3)	3.4343(1)
Bi1–Bi2$^{\#4}$	3.4476(3)	3.4343(1)
Bi2–Rh1$^{\#1}$	2.8299(2)	2.8204(1)
I2–Ag1	2.804(2)	2.7959(8)
I2–Ag2	2.854(4)	2.836(1)
I3–Ag2	2.8523(18)	2.8623(5)
I3–Ag1	2.924(2)	2.8997(8)
Ag1···Ag2	3.066(3)	3.0464(9)

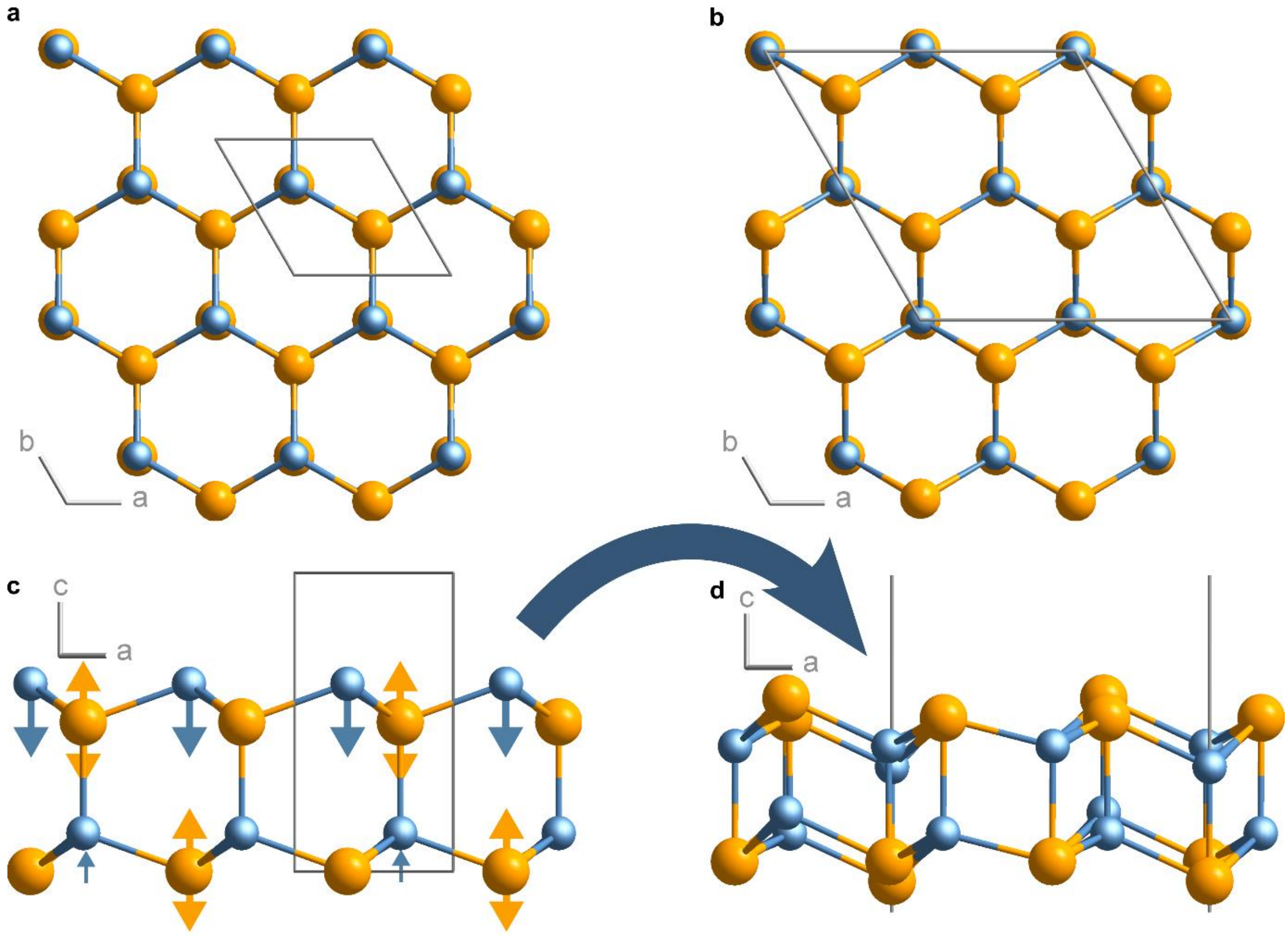

Figure S9. Comparison between a section of β-AgI (a,c) and the spacer in $Bi_{12}Rh_3Ag_6I_9$ (b, d). The latter has a 75% statistical occupancy of the tetrahedral voids. The similarity between the two structures is easily seen from the top view (a,b). In the spacer layer of $Bi_{12}Rh_3Ag_6I_9$ additional tetrahedral voids are filled, which can alternatively be regarded as a rearrangement of Ag^+ cations from neighboring sites. Arrows highlight the most obvious differences in atomic positions. In γ-AgI, the same layers as in β-AgI are found perpendicular to the <111> diagonals.

S4. Electronic structure

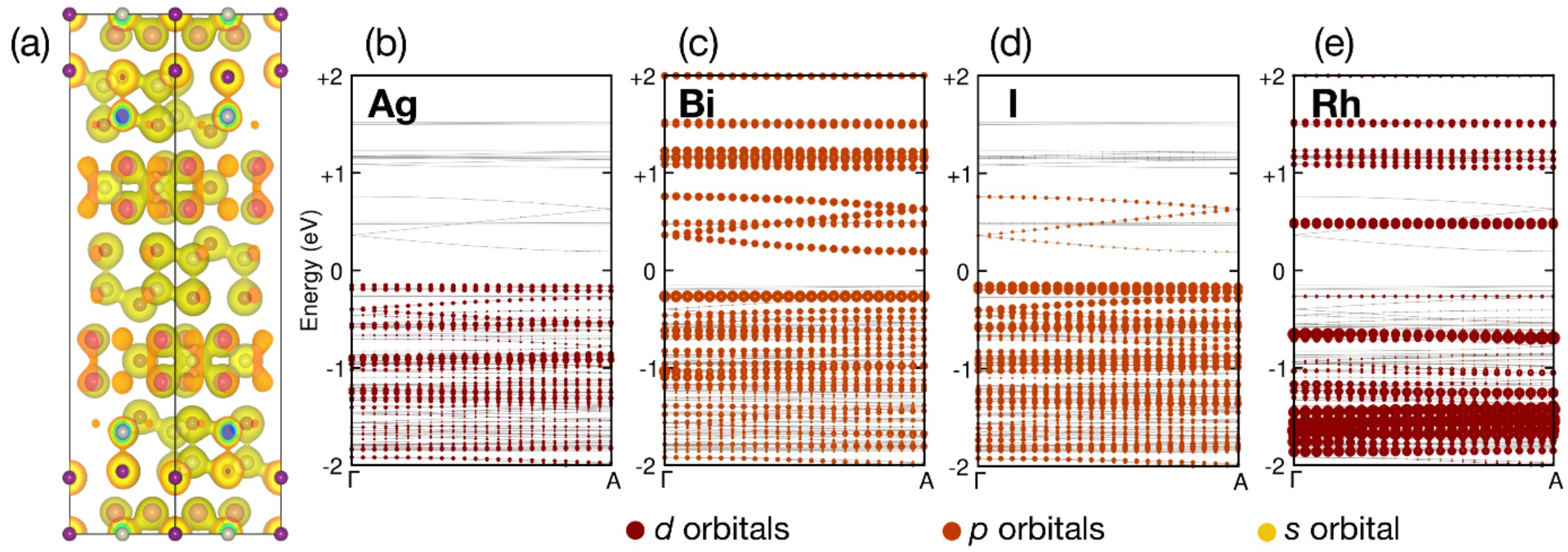

Figure S10. a) Charge density isosurface for $Bi_{12}Rh_3Ag_6I_9$. Note the weak interaction among spacer and intermetallic layers. The colors used for the isosurface are not related to the colors of next panels; b-d) Orbital and element-projected band structures along Γ-A path. *d* orbitals are represented by dark-red points, *p* orbitals by orange points and *s* orbitals by yellow ones.

Table S5. Structure parameters for the DFT calculations on $Bi_{12}Rh_3Ag_6I_9$.

```
1.0
 9.1585998535 0.0000000000 0.0000000000
 -4.5792999268 7.9315801362 0.0000000000
 0.0000000000 0.0000000000 38.9462013245
 Bi Rh I Ag
 36 9 27 18
Direct
 0.434599966 0.217299983 0.040839996
 0.565400004 0.782700002 0.959160030
 0.782700002 0.217299983 0.040839996
 0.217299983 0.782700002 0.959160030
 0.782700002 0.565400004 0.040839996
 0.217299983 0.434599966 0.959160030
 0.101266630 0.550633311 0.374173343
 0.232066706 0.116033353 0.292493343
 0.449366689 0.550633311 0.374173343
 0.883966625 0.116033353 0.292493343
 0.449366689 0.898733377 0.374173343
 0.883966625 0.767933309 0.292493343
 0.767933309 0.883966625 0.707506657
 0.898733377 0.449366689 0.625826657
 0.116033353 0.883966625 0.707506657
 0.550633311 0.449366689 0.625826657
 0.116033353 0.232066706 0.707506657
 0.550633311 0.101266630 0.625826657
 0.217369989 0.434739977 0.040609997
 0.782630026 0.565260053 0.959389985
 0.565260053 0.782630026 0.040609997
 0.434739977 0.217369989 0.959389985
 0.217369989 0.782630026 0.040609997
 0.782630026 0.217369989 0.959389985
 0.884036660 0.768073320 0.373943329
 0.449296683 0.898593366 0.292723328
```

0.231926695 0.115963347 0.373943329
0.101406641 0.550703347 0.292723328
0.884036660 0.115963347 0.373943329
0.449296683 0.550703347 0.292723328
0.550703347 0.101406641 0.707276642
0.115963347 0.231926695 0.626056671
0.898593366 0.449296683 0.707276642
0.768073320 0.884036660 0.626056671
0.550703347 0.449296683 0.707276642
0.115963347 0.884036660 0.626056671
0.499999970 0.000000000 0.000000000
0.000000000 0.499999970 0.000000000
0.500000000 0.500000000 0.000000000
0.166666642 0.333333343 0.333333343
0.666666687 0.833333313 0.333333343
0.166666701 0.833333373 0.333333343
0.833333313 0.666666687 0.666666687
0.333333343 0.166666642 0.666666687
0.833333373 0.166666701 0.666666687
0.000000000 0.000000000 0.000000000
0.666666687 0.333333343 0.333333343
0.333333343 0.666666687 0.666666687
0.000000000 0.000000000 0.108379990
0.000000000 0.000000000 0.891620040
0.666666687 0.333333343 0.441713333
0.666666687 0.333333343 0.224953339
0.333333343 0.666666687 0.775046647
0.333333343 0.666666687 0.558286667
0.162299991 0.324599981 0.212789983
0.837700009 0.675400019 0.787209988
0.675400019 0.837700009 0.212789983
0.324599981 0.162299991 0.787209988
0.162299991 0.837700009 0.212789983
0.837700009 0.162299991 0.787209988
0.828966677 0.657933295 0.546123326
0.504366696 0.008733352 0.120543353
0.342066675 0.171033338 0.546123326
0.991266668 0.495633334 0.120543353
0.828966677 0.171033338 0.546123326
0.504366696 0.495633334 0.120543353
0.495633334 0.991266668 0.879456639
0.171033338 0.342066675 0.453876674
0.008733352 0.504366696 0.879456639
0.657933295 0.828966677 0.453876674
0.495633334 0.504366696 0.879456639
0.171033338 0.828966677 0.453876674
0.160899982 0.321799964 0.137199983
0.839100003 0.678200006 0.862800002
0.678200006 0.839100003 0.137199983
0.321799964 0.160899982 0.862800002
0.160899982 0.839100003 0.137199983
0.839100003 0.160899982 0.862800002
0.827566624 0.655133307 0.470533311
0.505766690 0.011533369 0.196133345
0.344866693 0.172433347 0.470533311
0.988466620 0.494233310 0.196133345
0.827566624 0.172433347 0.470533311
0.505766690 0.494233310 0.196133345
0.494233310 0.988466620 0.803866625
0.172433347 0.344866693 0.529466689
0.011533369 0.505766690 0.803866625
0.655133307 0.827566624 0.529466689
0.494233310 0.505766690 0.803866625
0.172433347 0.827566624 0.529466689

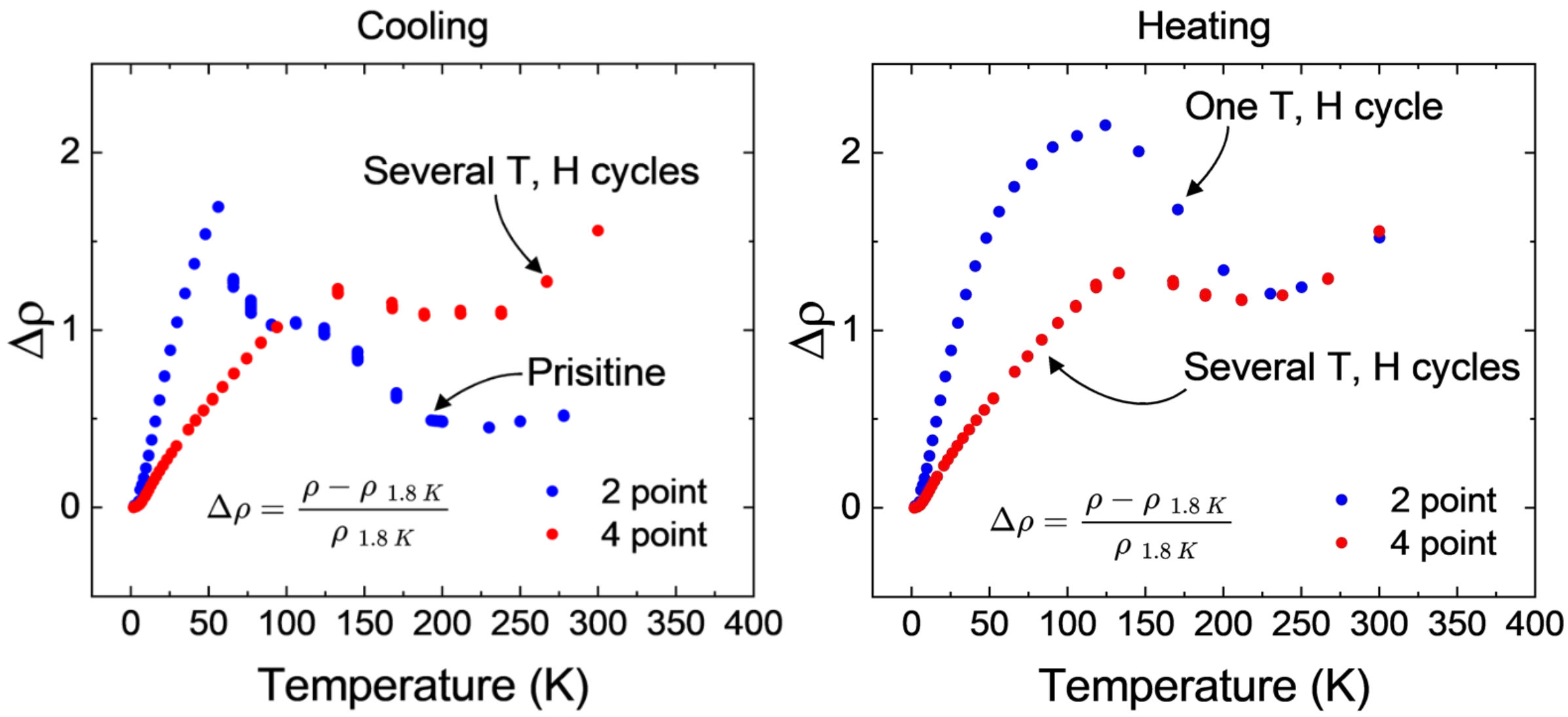

Figure S11. Comparison of normalized resistance data obtained from a two-point measurement on a pristine $Bi_{12}Rh_3Ag_6I_9$ crystal and from a four-point measurement on a pretreated crystal from the same batch. The same contacting material and attachment method were used in both cases. The two-point measurement captures the first temperature and current cycle of a pristine crystal, whereas the four-point measurement was performed after vacuum exposure, current flow, temperature and field cycling, and mechanical trimming to defined geometry. The cooling branches (left) therefore compare different initial states. The heating branches (right) compare conditioned states and show the same characteristic temperature dependence, including the broad maximum near 130 K. The remaining differences mainly reflect different sample geometries, contact configurations, and pretreatment histories.
